# Structural analysis of Cu/Zn‐superoxide dismutase linked to neurodegenerative disease by antibody‐guided cryo‐EM

**DOI:** 10.1002/pro.70615

**Published:** 2026-05-05

**Authors:** Yuki Shino, Norifumi Muraki, Yui Kobatake, Hiroaki Kamishina, Hirofumi Kosuge, Makoto Nakakido, Kouhei Tsumoto, Yoshiaki Furukawa

**Affiliations:** ^1^ Department of Chemistry Keio University Yokohama Japan; ^2^ Institute of Bioresource Engineering Ishikawa Prefectural University Nonoichi Japan; ^3^ Joint Department of Veterinary Medicine Faculty of Applied Biological Science, Gifu University Gifu Japan; ^4^ Center for One Medicine Innovative Translational Research (COMIT) Gifu University Gifu Japan; ^5^ Kyoto AR Advanced Veterinary Medical Center Kyoto Japan; ^6^ Department of Bioengineering, School of Engineering The University of Tokyo Tokyo Japan; ^7^ Department of Chemistry and Biotechnology, School of Engineering The University of Tokyo Tokyo Japan; ^8^ The Institute of Medical Science The University of Tokyo Tokyo Japan

**Keywords:** amyotrophic lateral sclerosis, antibody, canine degenerative myelopathy, cryo‐EM, Cu/Zn‐superoxide dismutase, protein misfolding

## Abstract

Accumulation of misfolded proteins is a hallmark of many neurodegenerative diseases. To characterize such misfolded species in vivo, conformation‐specific antibodies are widely used; however, limited knowledge of antibody–epitope interactions often hampers mechanistic insight. To address this, we determined the cryo‐electron microscopy structure of the complex between a monoclonal antibody, 19A9, and Cu/Zn‐superoxide dismutase (SOD1), a protein associated with canine degenerative myelopathy (DM), which is related to human amyotrophic lateral sclerosis. Biochemical analyses confirmed that 19A9 specifically recognizes monomeric SOD1, and the structure revealed binding near the interface normally used for homodimerization in native SOD1, with steric hindrance preventing interaction when the protein is in its homodimeric form. Immunofluorescence staining of spinal cord sections revealed that 19A9 stained a subset of motoneurons in DM‐affected dogs, but not in asymptomatic controls. Structural characterization of the 19A9‐monomeric SOD1 complex enabled us to propose that SOD1 monomers can arise in vivo under pathological conditions.

## 1 INTRODUCTION

Protein misfolding is a defining feature of many neurodegenerative diseases (Soto & Estrada, 2008). Misfolded proteins are proposed to exert toxicity through multiple mechanisms, including proteostasis impairment (Hipp et al., 2014), mitochondrial dysfunction (Israelson et al., 2010), and microglia‐mediated neuroinflammation (Gao et al., 2023). Because of such multifaceted toxicity, understanding how proteins misfold and adopt pathogenic conformations is essential not only for deciphering disease mechanisms but also for guiding the development of therapeutic strategies.

Various approaches have been employed to clarify how proteins misfold in vivo, including determination of atomic structures of fibrillar aggregates isolated from patient tissues (Todd et al., 2024) and analysis of protein folding in living cells using biophysical methods such as in‐cell nuclear magnetic resonance (NMR) spectroscopy (Luchinat & Banci, 2017). Conformation‐specific antibodies are also widely used, as they can selectively recognize non‐native structural states even when misfolded proteins are present only in small amounts, providing a practical means to detect and probe misfolded proteins under pathological conditions (De Genst et al., 2014). These antibodies are typically generated using antigens that expose normally buried regions or by immunizing with mutant or denatured proteins. Epitope mapping has offered clues about conformational changes; however, the structural information of epitopes has rarely been defined, making it difficult to link antibody reactivity to specific misfolded conformations in vivo.

Misfolding of mutant Cu/Zn‐superoxide dismutase (SOD1) in spinal motoneurons is a pathological hallmark of canine degenerative myelopathy (DM) (Awano et al., 2009), which shares genetic and pathological features of familial amyotrophic lateral sclerosis (ALS) (Rosen et al., 1993). Native SOD1 forms a homodimer, with each subunit coordinating one copper and one zinc ion and containing a conserved intramolecular disulfide bond (Tainer et al., 1982). Disruption of this native architecture is thought to underlie the misfolding process in vivo. To investigate how SOD1 misfolds in vivo, a variety of conformation‐specific antibodies have been generated (Furukawa & Tokuda, 2020; Rotunno & Bosco, 2013). One strategy has been to raise antibodies against peptide fragments corresponding to regions of the SOD1 primary sequence (Forsberg et al., 2010; Kerman et al., 2010; Rakhit et al., 2007). Such antibodies, often polyclonal, have been used to identify linear regions that become exposed during misfolding, and several have been reported to stain motor neurons in ALS patient tissues but not in healthy controls. In parallel, monoclonal antibodies have been generated by immunization with synthetic peptides corresponding to specific regions of mutant human SOD1 (Fujisawa et al., 2012; Xia et al., 2021), by immunization with the full‐length mutant protein (Gros‐Louis et al., 2010; Kobatake et al., 2017; Urushitani et al., 2007), or by isolating antibody clones directly from B cells of elderly individuals (Maier et al., 2018). These antibodies can distinguish abnormal conformations associated with mutant SOD1 while sparing the native dimeric structure. However, the precise conformations they detect remain undefined, limiting direct insight into how SOD1 misfolds in vivo. Previously, we addressed this challenge by determining the complex structure of the DM‐associated mutant canine SOD1 with the monoclonal antibody 22E1, thereby elucidating the mechanism by which 22E1 specifically suppresses SOD1 aggregation (Shino et al., 2024). Although this structural analysis with 22E1 allowed us to define the epitope and explain the mutation‐specific recognition, the corresponding biological observation—that the native form of SOD1^E40K^ is present in DM‐affected tissues—was not unexpected. Nonetheless, defining the structural basis of antibody recognition is essential not only for understanding antibody specificity but also for establishing antibodies as reliable tools to characterize protein conformations in vivo.

In this study, we determined the structure of SOD1 in complex with a conformation‐specific antibody by single‐particle cryo‐electron microscopy (cryo‐EM). We generated a monoclonal antibody, 19A9, using canine SOD1 carrying the E40K mutation (SOD1^E40K^), a variant associated with DM in dogs, as the antigen. 19A9 was found to bind exclusively to a monomerized SOD1; after being engineered into a trivalent antibody format, its complex with monomerized SOD1 was reconstructed at 2.95 Å resolution. The structure revealed that 19A9 engages a three‐dimensional region near the native dimer interface, confirming a conformational epitope. Steric hindrance prevents binding to the intact dimer, thereby explaining the strict monomer specificity of 19A9. Given that dissociation of SOD1 homodimer is proposed to occur on the pathway to misfolding and aggregation, we assessed the pathological relevance of monomeric SOD1 by immunofluorescence staining of canine spinal cord tissues and found that 19A9 labeled a subset of motoneurons in certain DM‐affected dogs, but not in asymptomatic controls. Grounded in our detailed structural characterization, these findings suggest that SOD1 monomers can arise in the pathological conditions in vivo. More broadly, this study demonstrates a versatile approach for defining the structural basis of misfolded proteins in neurodegenerative diseases.

## 2 RESULTS

### 2.1 Isolation of a monoclonal antibody raised against mutant SOD1 and selectively recognizing its monomeric form

**FIGURE 1:**
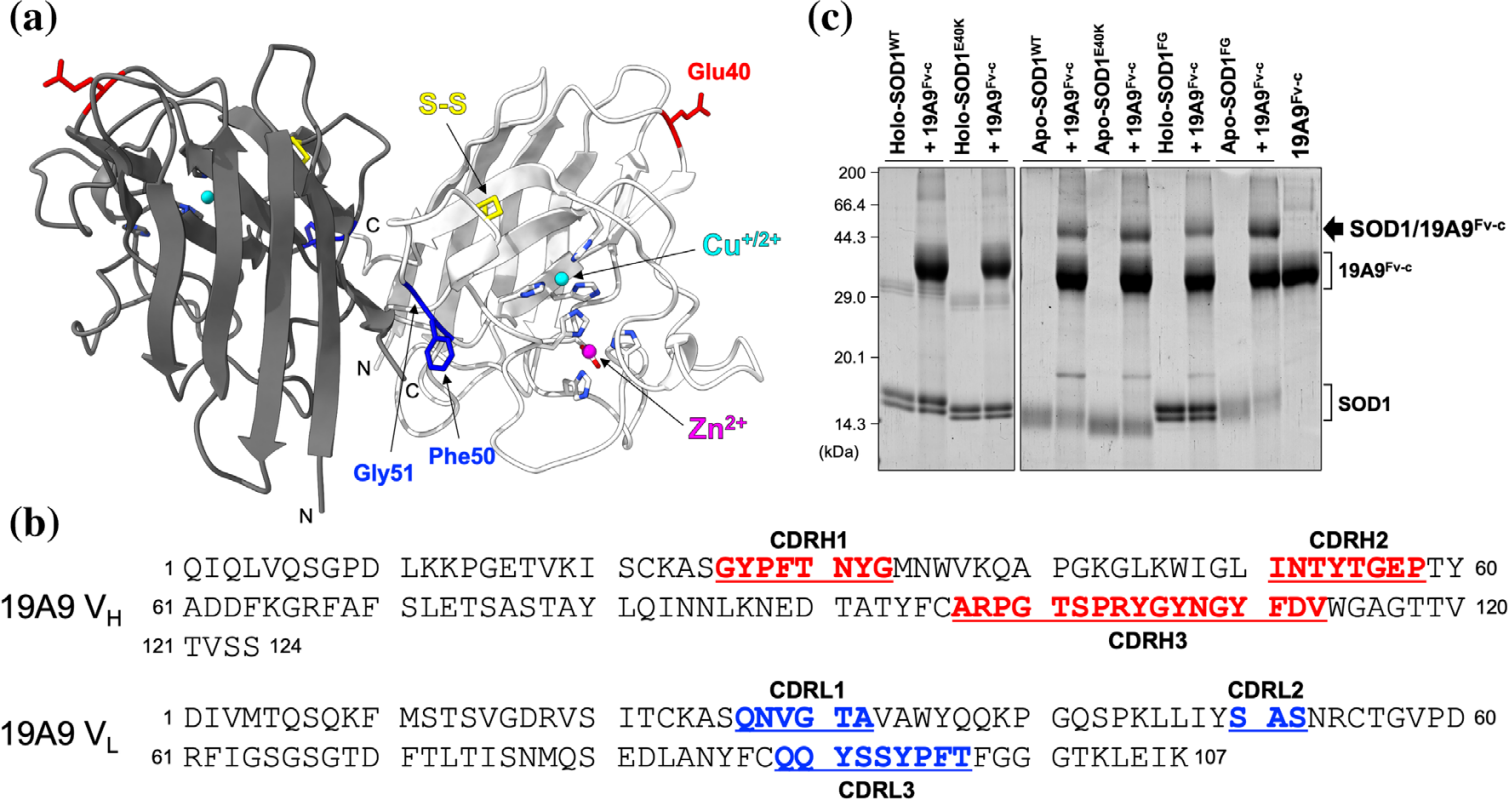
A monoclonal antibody 19A9 binds to monomeric superoxide dismutase (SOD1) (a) Crystal structure of wild‐type canine SOD1 (PDB ID: 7WWT). A copper ion (cyan sphere) and a zinc ion (magenta sphere) are shown together with their coordinating ligands in stick representation. The intra‐subunit disulfide bond between Cys57 and Cys146 is depicted in yellow. Glu40 and Phe50/Gly51 are highlighted in red and blue, respectively. (b) Amino acid sequences of the V_H_ and V_L_ domain (V‐J region) of 19A9. The three complementarity‐determining regions (CDRH1‐3 for V_H_ and CDRL1‐3 for V_L_), as identified with IMGT/V‐QUEST, are highlighted in red (V_H_) and blue (V_L_). (c) Chemical crosslinking assay with the amine‐reactive crosslinker bis(sulfosuccinimidyl)suberate, used to examine the binding of SOD1 variants to 19A9^Fv‐c^. Holo‐SOD1^WT/E40K/FG^ denotes proteins that bind a copper and zinc ion and contain the conserved intramolecular disulfide bond. Apo‐SOD1^WT/E40K^ lacks both metal ions and the disulfide bond, whereas apo‐SOD1^FG^ likewise lacks the metal ions but retains the disulfide bond.

As described in Section 1, DM is a neurodegenerative disease in dogs caused by mutant SOD1, sharing genetic and pathological features with human ALS (Awano et al., 2009). We previously generated monoclonal antibodies by using the pathogenic E40K mutant form of SOD1 (SOD1^E40K^) as the antigen (Kobatake et al., 2017) (Figure 1a). Among those confirmed to bind to glutathione S‐transferase‐fused SOD1^E40K^ by enzyme‐linked immunosorbent assay, we chose clone 19A9 in this study and examined its specificity.

To evaluate the binding specificity of 19A9 with tag‐free SOD1 proteins, the variable heavy (V_H_) and light (V_L_) domains of 19A9 were sequenced (Figure 1b). The three complementarity‐determining regions (CDRs)—CDRH1‐3 in V_H_ and CDRL1‐3 in V_L_—were identified. These hypervariable loop segments form the antigen‐binding site and determine binding specificity. The V_H_ and V_L_ of 19A9 were then engineered into a compact antibody fragment called Fv‐clasp (19A9^Fv‐c^), in which each V_H_ and V_L_ domain was fused at the C‐terminus to a SARAH (Salvador/RASSF1A/Hippo) domain (approximately 50‐residue small helical domain) derived from human Mst1 (Arimori et al., 2017). The SARAH domains promote stable heterodimerization between V_H_ and V_L_, mimicking the antigen‐binding structure of a full‐length antibody while enabling efficient large‐scale production, which is advantageous for rigorous biochemical evaluation of its interaction with untagged SOD1 proteins.

Given that the conformation of SOD1 is influenced by its metal‐binding and thiol‐disulfide status (Arnesano et al., 2004) (Figure 1a), we first employed the amine‐reactive crosslinker bis(sulfosuccinimidyl)suberate to broadly assess which conformational states of SOD1 are recognized by 19A9^Fv‐c^. As shown in Figure 1c, neither wild‐type SOD1 (SOD1^WT^) nor SOD1^E40K^ with the disulfide bond in the holo state (with a copper and zinc ion bound) was crosslinked with 19A9^Fv‐c^. In contrast, both SOD1^WT^ and SOD1^E40K^ in the apo (without a copper and zinc ion bound) and disulfide‐reduced state exhibited a clear band corresponding to a crosslinked complex with 19A9^Fv‐c^. It is also notable that holo‐SOD1 exhibits two distinct bands at approximately 15 kDa upon crosslinking, likely reflecting discrete intramolecularly crosslinked species, whereas apo‐SOD1 shows more diffuse bands due to its greater conformational flexibility. SOD1 is known to exist predominantly as a stable homodimer in the holo form (Figure 1a), but undergoes monomerization in the apo and disulfide‐reduced state (Arnesano et al., 2004). Thus, our crosslinking data suggest that 19A9 selectively recognizes monomeric SOD1. Supporting this notion, an artificially monomerized variant of SOD1 carrying F50E/G51E mutations (SOD1^FG^) (Bertini et al., 1994) (Figure 1a) with the intramolecular disulfide bond was efficiently crosslinked by 19A9^Fv‐c^ in both the holo and apo state (Figure 1c).

**FIGURE 2:**
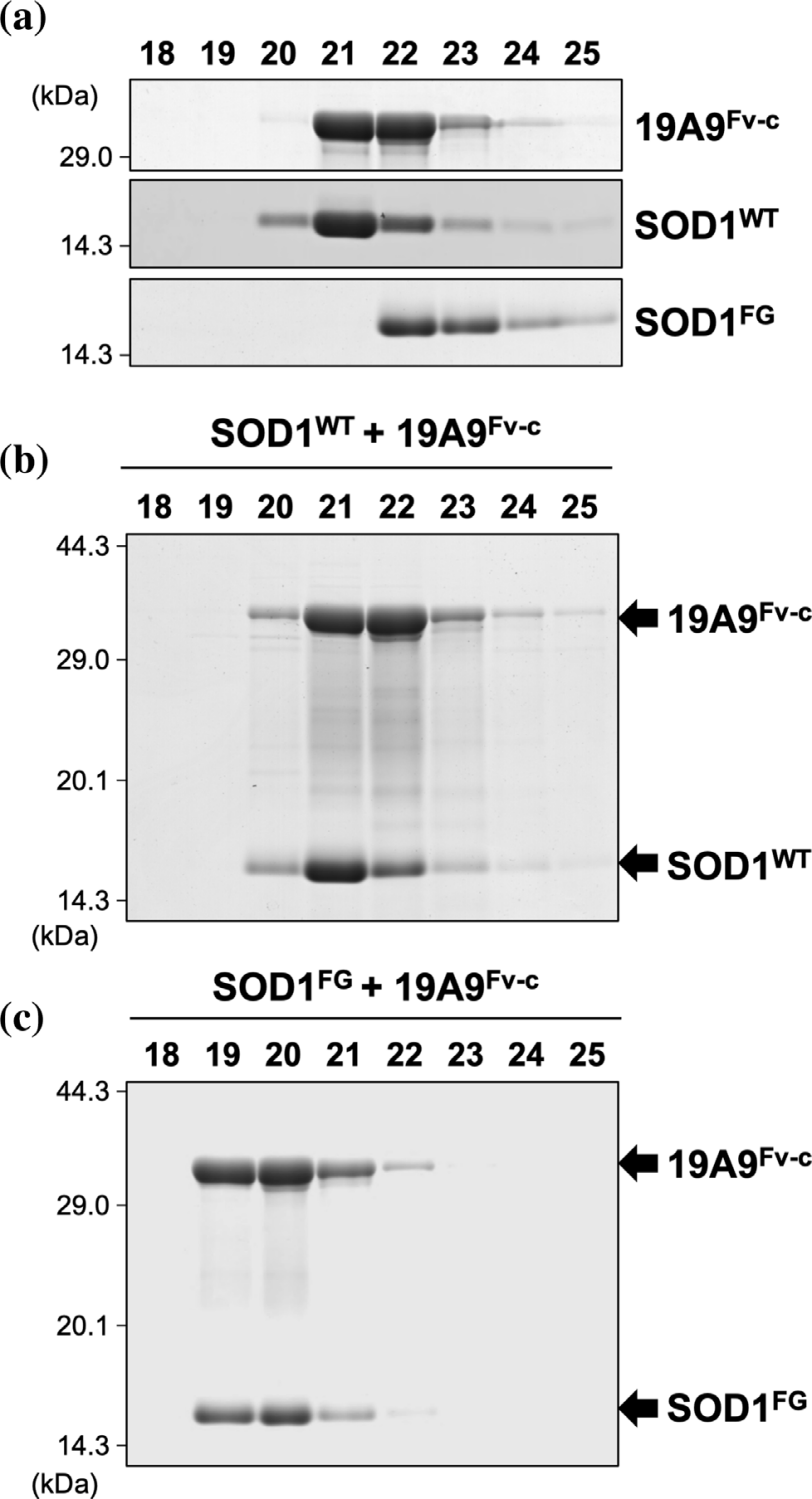
Examination of 19A9^Fv‐c^ binding to monomeric superoxide dismutase (SOD1) by size‐exclusion chromatography (a) Individual proteins (19A9^Fv‐c^, holo‐SOD1^WT^, and holo‐SOD1^FG^; 10 μM) were analyzed by gel‐filtration. The numbers indicated above each lane represent the fraction numbers, which correspond directly to the elution time (in minutes), as fractions were collected at 1‐min intervals. Fractions 18–25 were examined by non‐reducing SDS‐PAGE followed by Coomassie brilliant blue staining. (b, c) 19A9^Fv‐c^ (10 μM) was mixed with equimolar holo‐SOD1^WT^ (b) or holo‐SOD1^FG^ (c) and analyzed as described in (a).

Selective recognition of monomeric SOD1 by 19A9^Fv‐c^ was further validated by size‐exclusion chromatography. As shown in Figure 2a, 19A9^Fv‐c^ and holo‐SOD1^FG^ were individually eluted at fraction numbers 21/22 and 22/23, respectively. When an equimolar mixture of 19A9^Fv‐c^ and holo‐SOD1^FG^ was analyzed, both proteins were co‐eluted earlier than when run separately, appearing together at fraction numbers 19/20 (Figure 2c). This shift indicates complex formation between SOD1^FG^ and 19A9^Fv‐c^. In contrast, holo‐SOD1^WT^, which exists predominantly as homodimers, was eluted slightly earlier than monomeric SOD1^FG^, at fraction numbers 21/22 (Figure 2a). Importantly, the elution profiles of SOD1^WT^ and 19A9^Fv‐c^ remained unchanged when mixed, suggesting that 19A9^Fv‐c^ does not bind to dimeric SOD1 (Figure 2b). Specific binding of full‐length 19A9 IgG to holo‐SOD1^FG^, but not to holo‐SOD1^WT^, was also confirmed by size‐exclusion chromatography (Figure S1). Together, these results demonstrate that 19A9 IgG and its fragment antibody, 19A9^Fv‐c^, selectively recognize the monomeric conformation of SOD1.

### 2.2 Structural analysis of the 19A9‐monomeric SOD1 complex

To elucidate the structural basis for the specific recognition of monomeric SOD1 by 19A9, we first attempted to determine the crystal structure of the 19A9^Fv‐c^‐SOD1^FG^ complex. Despite screening approximately 900 crystallization conditions, however, we were unable to obtain crystals of the complex. This likely reflects the conformational flexibility of monomeric SOD1^FG^, which may hinder crystal lattice formation. We therefore turned to single‐particle cryo‐EM, which enables structural analysis without the need for crystallization.

To facilitate the analysis with cryo‐EM, which benefits from molecular symmetry, we engineered a trivalent antibody fragment (triabody) by directly linking the V_H_ and V_L_ domains of 19A9 (designated 19A9^Tri^). Three such V_H_–V_L_ units associate intermolecularly in an alternating manner to form a stable trimer with C3 symmetry (Pei et al., 1997). Imposing C3 symmetry facilitates cryo‐EM reconstruction by effectively increasing the signal‐to‐noise ratio through averaging of equivalent views. Importantly, we confirmed that the specificity of 19A9 for monomeric SOD1 was preserved in the triabody format. 19A9^Tri^ was eluted at fraction numbers 23/24, slightly later than monomeric SOD1^FG^ (fraction numbers 22/23) (Figure S2A). Upon mixing 19A9^Tri^ with SOD1^FG^, both proteins were co‐eluted significantly earlier at fraction numbers 18/19, consistent with complex formation (Figure S2C). In contrast, mixing with dimeric SOD1^WT^ did not alter the elution profile of 19A9^Tri^, indicating no detectable interaction (Figure S2B).

**FIGURE 3:**
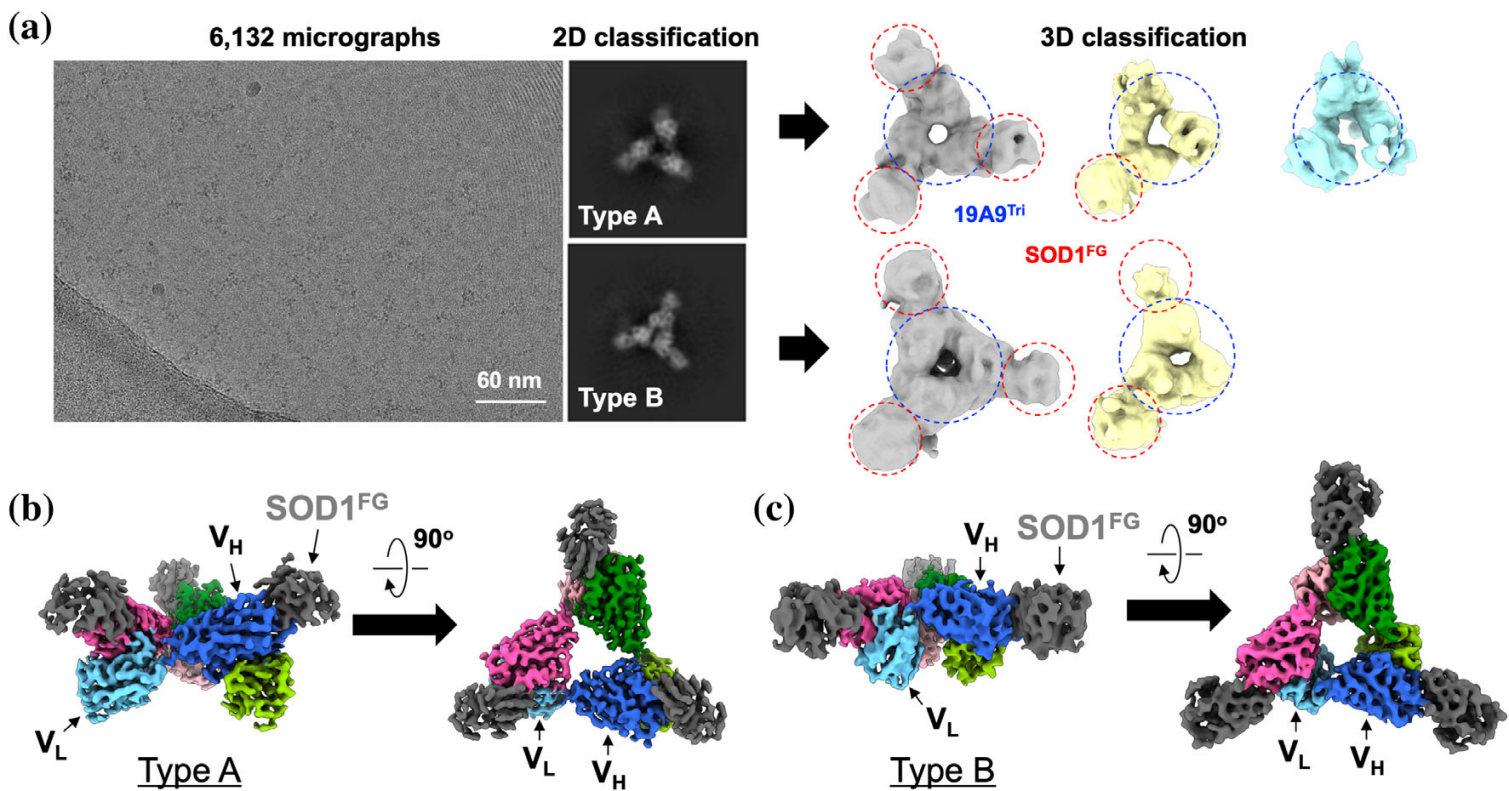
Reconstruction of the SOD1^FG^‐19A9^Tri^ complex by cryo‐electron microscopy (cryo‐EM) (a) Representative cryo‐EM micrograph of SOD1^FG^‐19A9^Tri^ particles (left). Particle images were subjected to 2D classification, which revealed two major particle types, type A and type B, illustrated by representative classes (middle). Subsequent 3D classification of type A and type B particles yielded reconstructions showing the triangular 19A9^Tri^ scaffold (blue dotted circles) with and without SOD1^FG^ bound (red dotted circles). (b, c) Cryo‐EM density maps of the type A (b) and type B (c) complex are shown at thresholds of 0.490 and 0.280, respectively. A triabody is composed of three V_H_‐V_L_ units, colored blue, pink, and green, with V_H_ shown in a darker shade and V_L_ in a lighter shade of the corresponding color. SOD1^FG^ is shown in dark gray. SOD1, superoxide dismutase.

To optimize the distribution of 19A9^Tri^/SOD1^FG^ particles on cryo‐EM grids, the complex was purified by size‐exclusion chromatography (~10 mg/mL) and supplemented with fluorinated Fos‐Choline‐8 (1 mM final). In micrographs, particles displayed the triangular shape characteristic of the 19A9^Tri^ scaffold (Figure 3a, left). As detailed in Section 4 and Figure S3, particle images were subjected to two‐dimensional (2D) classification into multiple classes. Examination of these classes revealed two predominant particle types: one with a larger triangular shape (type A) and another with a smaller triangular shape (type B) (Figure 3a, middle). Accordingly, three‐dimensional (3D) classification was performed separately for type A and type B.

For type A, three reconstructions were obtained (Figure 3a, right): one that likely represented a complete complex with three SOD1^FG^ molecules bound to the triangular 19A9^Tri^ scaffold, and two that appeared to correspond to incomplete complexes with one or no SOD1^FG^ molecules bound. Because the latter two reconstructions were of insufficient resolution to resolve secondary structures, they were not further analyzed. For type B, two reconstructions were obtained that appeared to correspond to a complete complex with three bound SOD1^FG^ molecules and an incomplete complex with one (or two) SOD1^FG^ (Figure 3a, right).

To maximize particle recovery, particles from the complete‐complex reconstruction were used to train the cryoSPARC Deep Picker model, which was then applied to all micrographs to recover additional corresponding particles. This yielded high‐resolution cryo‐EM density maps of the complete complexes, resolved to global resolutions of 2.95 and 3.91 Å (Fourier shell correlation (FSC) gold standard = 0.143) for type A and type B, respectively (Figure 3b,c). Owing to their triangular configuration, the V_H_ and V_L_ domains were readily identifiable in these maps. Moreover, densities associated with this triangular scaffold displayed β‐sheet features characteristic of SOD1.

**FIGURE 4:**
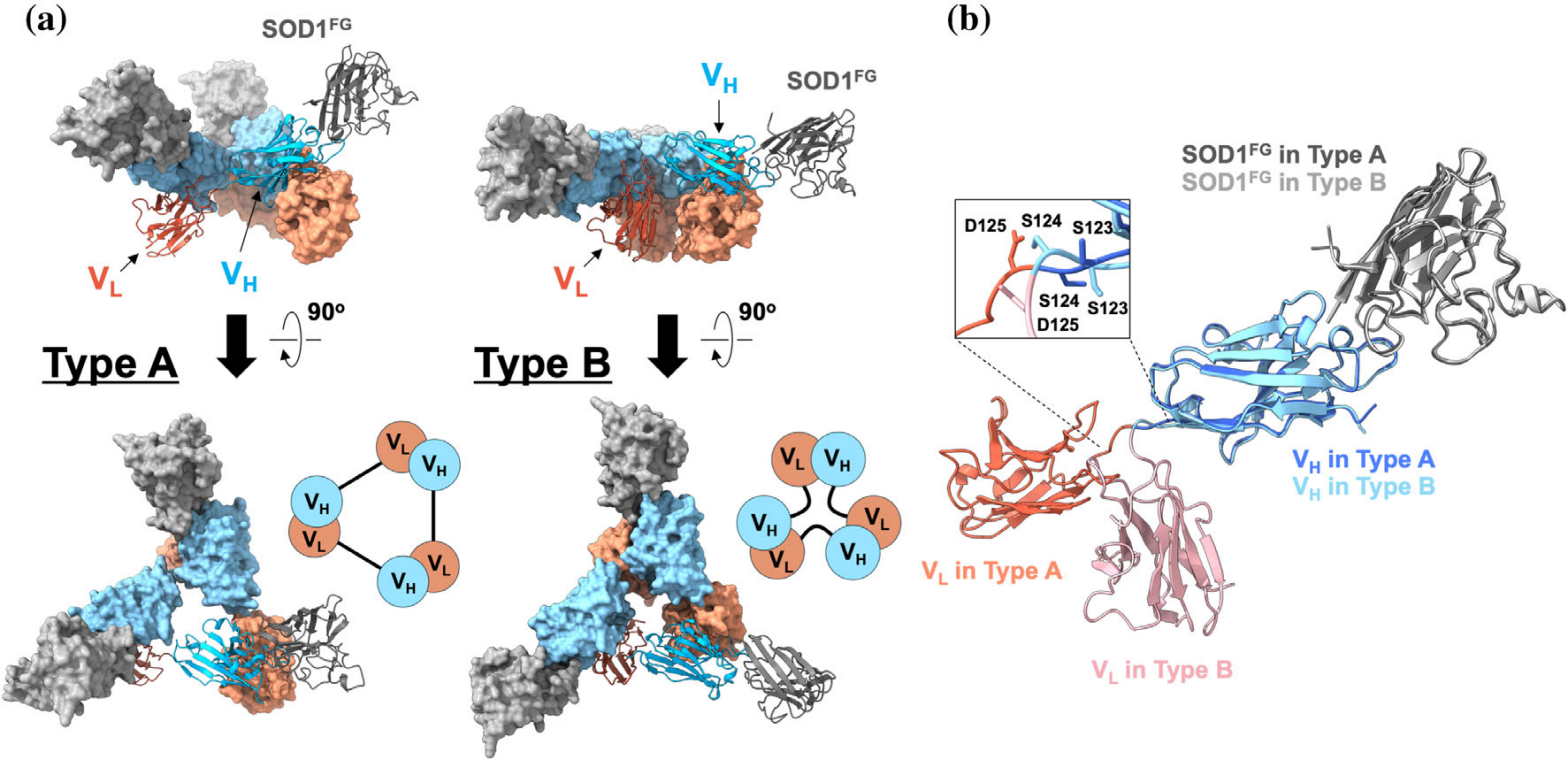
Overall structure of the SOD1^FG^‐19A9^Tri^ complex (a) Structural models of the SOD1^FG^‐19A9^Tri^ complex (PDB ID: 21EN and 21EO for type A and type B, respectively) were built based on the density maps shown in Figure 3b,c. For clarity, one unit consisting of a single 19A9^Tri^ molecule (V_H_ in dark sky blue, V_L_ in tomato) bound to a SOD1^FG^ molecule (dark gray) is shown in cartoon representation, while the other 2 units are shown in surface representation (V_H_ in light sky blue, V_L_ in light salmon). Schematic representations of the configurations of the three V_H_‐V_L_ units in the triabody (types A and B) are also shown. (b) A unit of the type A complex was superimposed with the corresponding unit of the type B complex by aligning the SOD1^FG^ molecules. The V_H_‐V_L_ connecting region is enlarged in the inset. Coloring is as indicated in the figure. SOD1, superoxide dismutase.

Model building was performed using ColabFold‐predicted structures of the 19A9 V_H_–V_L_ complex and SOD1^FG^ as initial models, which were manually fitted into the cryo‐EM density maps and subsequently adjusted (see Section 4, Figure 4a). Because the CDR loops of V_H_ and V_L_ differ significantly in length (Figure 1b), the two domains could be readily distinguished in the density map. As illustrated in Figure 4b, the principal structural difference between the type A and type B complexes arises from the relative orientation of V_H_ and V_L_ within an individual 19A9^Tri^ molecule. In the 19A9^Tri^ scaffold, three 19A9^Tri^ molecules assemble into a triangular architecture in which each V_H_ interacts with the V_L_ of a neighboring molecule (Figure 4a), and SOD1^FG^ binds specifically to the V_H_ domain (vide infra). Importantly, SOD1^FG^ and its local arrangement with the V_H_ domain and its paired V_L_ are essentially conserved between type A and type B, indicating that the distinction between the two conformers reflects only the intramolecular V_H_–V_L_ configuration. Owing to its higher resolution, therefore, the type A complex structure was selected for subsequent analyses.

### 2.3 Specific recognition of monomeric SOD1 with 19A9

**FIGURE 5:**
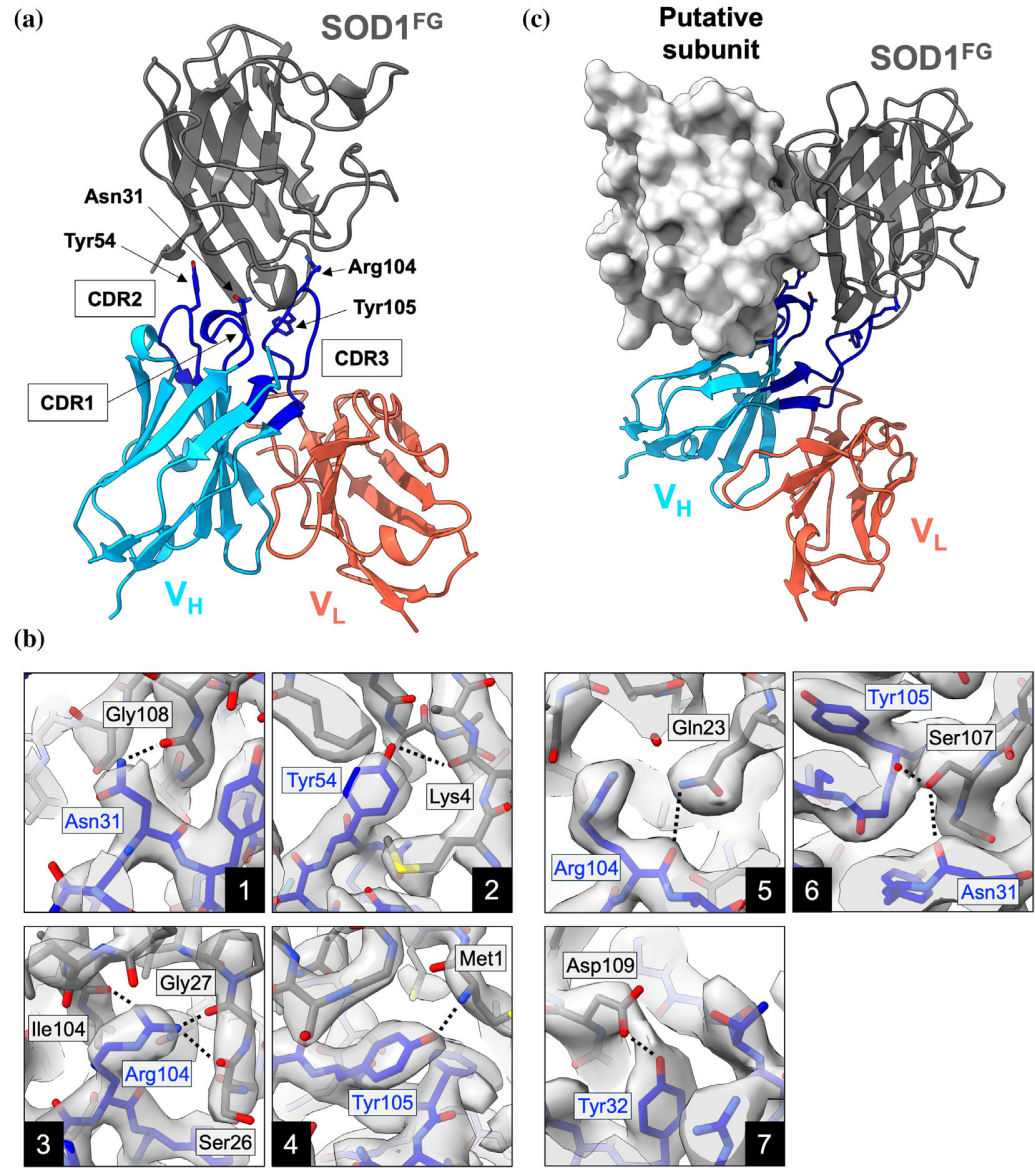
Interactions underlying the specific recognition of monomeric superoxide dismutase (SOD1) by 19A9 (a) A representative assembly of SOD1^FG^ (dark gray) bound to the V_H_ domain (dark sky blue) and its paired V_L_ domain (tomato) (PDB ID: 21EN). The three V_H_ complementarity‐determining regions (CDRs) (CDR1‐3) are highlighted in blue, and residues within these CDRs that interact with SOD1^FG^ are shown in stick representation. (b) Close‐up views of the interactions between V_H_ and SOD1^FG^. Residue labels are shown in blue (V_H_) and black (SOD1^FG^). All residues are displayed as sticks, with the cryo‐electron microscopy density map displayed as a surface. Dotted lines indicate hydrogen‐bonding interactions. (c) Structural basis for monomer specificity. One subunit of homodimeric SOD1^WT^ (PDB ID: 1HL5) is superimposed on SOD1^FG^ within the assembly of V_H_ (dark sky blue; CDRs in blue), V_L_ (tomato), and SOD1^FG^ (dark gray). The second subunit of the dimer, shown as a surface (indicated as “putative subunit”), sterically clashes with V_H_, explaining why 19A9 selectively recognizes monomeric rather than dimeric SOD1.

As shown in Figure 5a, 19A9^Tri^ binds to the SOD1 monomer primarily through its V_H_ domain, with minimal contribution from the V_L_ domain. In the complex, the three CDRs of the V_H_ domain engage the SOD1 monomer in an orientation that would sterically clash with the second subunit in the native homodimer, thereby precluding binding to dimeric SOD1 (Figure 5c). This provides a molecular explanation for the selective recognition of monomeric, but not homodimeric, SOD1 by 19A9.

The interactions between the V_H_ domain of 19A9 and the SOD1 monomer are mediated by multiple hydrogen bonds involving both side‐chain and main‐chain atoms of the V_H_ CDRs and SOD1 (Table S1). As summarized in Figure 5b, Asn31 of V_H_ forms a hydrogen bond with the backbone carbonyl of Gly108 in SOD1 (panel 1); Tyr54 of V_H_ interacts with the backbone carbonyl of Lys4 in SOD1 (panel 2); Arg104 of V_H_ forms hydrogen bonds with the backbone carbonyls of Ser26, Gly27, and Ile104 in SOD1 (panel 3); and Tyr105 of V_H_ engages the N‐terminal amino group of SOD1 (panel 4). In addition, hydrogen bonds are also observed between main‐chain atoms of V_H_ and side‐chain atoms of SOD1 (Figure 5b), including interactions between the carbonyl of Arg104 and the side chain of Gln23 (panel 5), and between the side chain of Ser107 and the carbonyls of Asn31 and Tyr105 (panel 6). A side chain–side chain interaction between Asp109 of SOD1 and Tyr32 of V_H_ is also present (Figure 5b, panel 7).

To experimentally assess the contribution of those V_H_ residues to SOD1 binding, we generated 19A9^Fv‐c^ variants in which each residue was substituted with alanine (N31A, Y54A, R104A, and Y105A), and evaluated their binding to SOD1^FG^ by size‐exclusion chromatography. As shown in Figure S4, the N31A and Y54A variants of 19A9^Fv‐c^ co‐eluted with SOD1^FG^ at fraction number 20, indicating that their ability to form a complex with SOD1^FG^ was retained. In contrast, the R104A and Y105A variants eluted later at fraction numbers 21/22, showing a loss of binding activity toward SOD1^FG^. These results demonstrate that Arg104 and Tyr105 of the V_H_ make critical contributions to complex formation with the SOD1 monomer.

It is noteworthy that the N‐terminal amino group of SOD1 is acetylated in humans (Barra et al., 1980) and is therefore likely to be acetylated in dogs as well, raising the possibility that such modification could influence the hydrogen‐bonding interaction between Tyr105 and the N‐terminal region of SOD1 (Figure 5b, panel 4). To address this, we examined N‐terminally acetylated SOD1, which had been monomerized by demetallation and disulfide reduction (Arnesano et al., 2004). As shown in Figure S5, the acetylated, disulfide‐reduced apo‐SOD1, which was expected to adopt a monomeric state, was successfully crosslinked with 19A9^Fv‐c^ but not with the Y105A variant of 19A9^Fv‐c^, indicating that N‐terminal acetylation does not interfere with the interaction between SOD1 and 19A9.

The V_H_–V_L_ arrangement in the complex adopts a canonical architecture commonly observed across antibodies, as exemplified by the Fab of HyHEL10—a mouse monoclonal IgG against hen egg white lysozyme that has served as a classical model in structural immunology (Acchione et al., 2009) (Figure S6A)—with an RMSD of 0.559 Å. Their relative orientation is largely stabilized though interactions in the framework regions of the V_H_ and V_L_. Interactions between V_H_ and V_L_ CDR3 loops are also commonly observed, but their detailed configurations vary among antibodies. As shown in Figure S6B, in 19A9, the side chain of Gln88 in V_L_ CDR3 forms a hydrogen bond with the backbone carbonyl of Gly109 in V_H_ CDR3, and Tyr90 and Phe95 of V_L_ CDR3 engage in hydrophobic contacts with Gly109 and Tyr110 of V_H_ CDR3. Residue numbering for V_L_ in 19A9^Tri^ is given independently, restarting from 1 at the N‐terminus of V_L_ (Figure 1b). In the Protein Data Bank (PDB) entry, residues are numbered sequentially from the N‐terminal Met across the fusion protein. Thus, Gln88, Tyr90, and Phe95 described above correspond to Gln214, Tyr216, and Phe221 in the deposited 19A9^Tri^ structure, respectively. Although V_L_ itself does not make direct contacts with SOD1, it may indirectly contribute to stable binding by reinforcing the V_H_‐SOD1 interaction through V_H_‐V_L_ contacts.

### 2.4 Structural features of monomeric SOD1 recognized by 19A9

**FIGURE 6:**
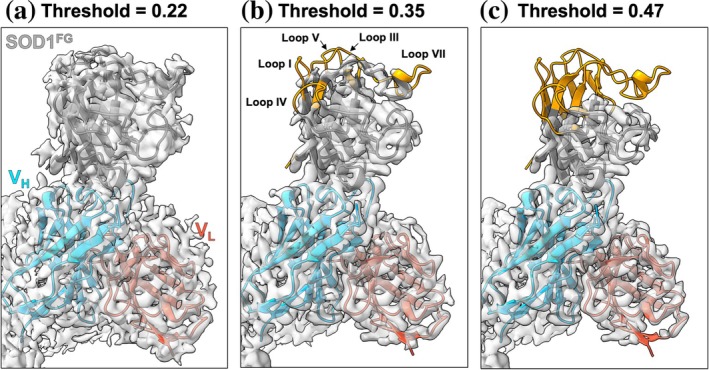
Visualization of structural flexibility in SOD1^FG^ by threshold‐dependent cryo‐electron microscopy (cryo‐EM) density maps. Cryo‐EM density maps of the SOD1^FG^‐19A9^Tri^ complex (PDB ID: 21EN) are shown at contour thresholds of (a) 0.22, (b) 0.35, and (c) 0.47. The backbone of the SOD1^FG^ model is displayed in cartoon representation, overlaid on the density maps in surface representation. At the lower threshold (a), the backbone is largely embedded within the density. As the threshold is increased (b, c), density is progressively lost in specific regions, exposing the underlying backbone. These regions correspond to flexible parts of SOD1^FG^, indicating higher structural fluctuations compared with the V_H_‐interacting surface. SOD1, superoxide dismutase.

In the 19A9^Tri^‐SOD1^FG^ complex, the modeled structure of SOD1^FG^ closely aligned with that of a single subunit of the SOD1^WT^ dimer, with a root‐mean‐square deviation (RMSD) of 0.919 Å. Whereas the 19A9^Tri^ scaffold was resolved to ~2.5 Å, however, SOD1^FG^ exhibited lower local resolution (~3.5 Å). Notably, the cryo‐EM density of SOD1^FG^ gradually weakened with increasing distance from the binding interface with 19A9^Tri^, suggesting enhanced structural fluctuation of SOD1 in regions distal to the interface. For a more quantitative assessment, density maps were visualized at different contour thresholds. At a threshold of 0.22, density was observed across the entire SOD1^FG^ region (Figure 6a). When the threshold was increased to 0.35, density was lost for residues 10–15 (Loop I), 37–43 (Loop III), 54–58 (Loop IV, often called as S‐S subloop), 88–94 (Loop V), 124–133 (Loop VII, often called as electrostatic loop), 145–146, and 152–153 (C‐terminal β8 strand) (Figure 6b). These regions of missing density expanded further when the threshold was raised to 0.47 (Figure 6c). Notably, the regions that lost density correspond to flexible loop regions on the hemisphere of SOD1 opposite to the V_H_‐binding surface. Although it remains unclear whether the observed asymmetric flexibility between the V_H_‐interacting hemisphere and the opposite hemisphere is an intrinsic property of monomeric SOD1 or a consequence of stabilization upon antibody binding, a NMR study has shown that Loops I and IV are intrinsically flexible in monomerized human SOD1^FG^ (Banci et al., 2000). The precise degree of folding or misfolding of SOD1 species recognized by 19A9 cannot be fully defined from the current data. Nevertheless, 19A9 binds a monomeric SOD1 conformation that retains a subunit‐like three‐dimensional architecture, rather than extensively unfolded or linearized forms, despite enhanced structural fluctuations. These findings highlight the ability of 19A9 to selectively detect monomeric SOD1, providing a reliable tool for probing this species in disease‐relevant in vivo contexts.

### 2.5 Monomeric SOD1 in spinal motoneurons of dogs with DM

**FIGURE 7:**
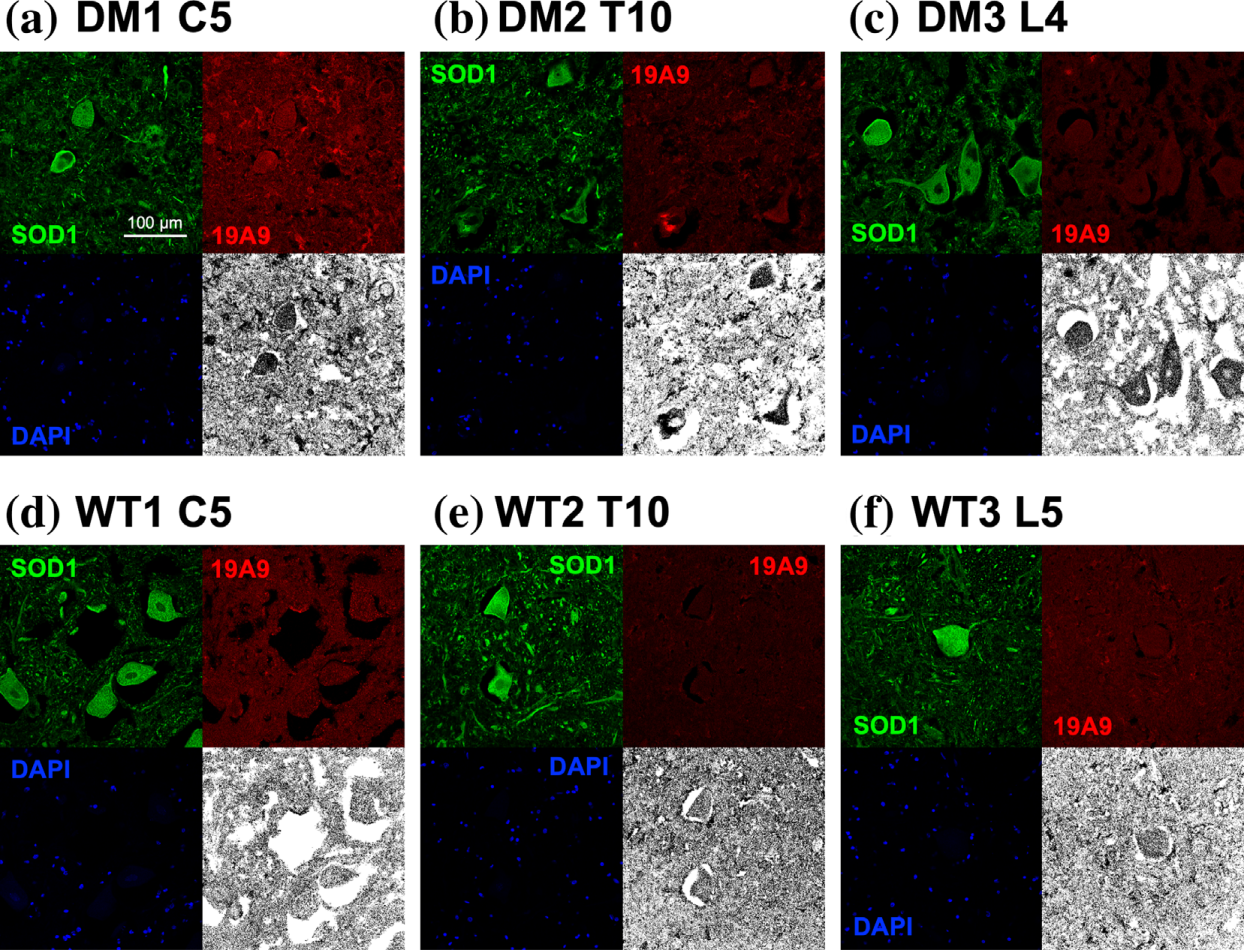
Detection of monomeric superoxide dismutase (SOD1) in motoneurons of degenerative myelopathy (DM)‐affected dogs by 19A9 double fluorescence immunohistochemistry was performed on cervical (C5), thoracic (T10), and lumbar (L4, L5) spinal cord sections from (a–c) three DM‐affected Pembroke Welsh Corgis (E40K homozygotes, DM1‐3) and (d–f) three asymptomatic Beagles (WT homozygotes, WT1‐3). Details of the animals are provided in Table S2. Sections were co‐stained with polyclonal anti‐SOD1 antibody (green) and recombinant monoclonal antibody 19A9 (red), with nuclear counterstaining by 6‐diamidino‐2‐phenylindole (DAPI) (blue). Grayscale images in the lower‐right corner of each panel show the 19A9 signal in the red channel after adjustment of the lower intensity threshold using ImageJ, which improved discrimination between stained and unstained cells.

Building on our structural validation of 19A9's specific recognition of monomeric SOD1, we examined spinal cord tissues of dogs using immunofluorescence staining with 19A9 in parallel with a polyclonal anti‐SOD1 antibody (SOD1 pAb). We analyzed cervical, thoracic, and lumbar spinal cord sections from DM‐affected as well as control dogs (WT) (Table S2). Despite the limited and unbalanced sample composition, Pembroke Welsh Corgis (PWC) were used for the disease group due to the high prevalence of SOD1‐associated DM in this breed (Chang et al., 2013; Zeng et al., 2014), whereas Beagles were used as controls because they are the only laboratory dogs readily available in Japan. As shown in Figures 7 and S7, motoneurons were robustly stained with SOD1 pAb, consistent with the known expression of SOD1 in these cells (Pardo et al., 1995). In contrast, staining with 19A9 was extremely faint, making it difficult to determine whether motoneurons were labeled. We thus applied thresholding to 19A9‐stained images, which improved discrimination between stained and unstained cells (the grayscale images shown in the lower‐right corner of each panel in Figures 7 and S7). This revealed the staining with 19A9 of the SOD1 pAb‐positive motoneurons in the spinal cord sections of the DM‐affected dogs (Figure 7a–c), while not all of the SOD1 pAb‐positive motoneurons of the DM‐affected dogs were clearly stained with 19A9 (e.g., Figure S7B,N,P). In contrast, spinal motoneurons of control dogs showed no detectable staining with 19A9 even after thresholding (Figures 7d–f and S7R–Y). Although the staining with 19A9 could not be evaluated quantitatively, these findings suggest that monomeric SOD1 species are present at trace levels in a subset of motoneurons in DM‐affected dogs but are below detectable levels in controls.

## 3 DISCUSSION

In this study, we generated a monoclonal antibody, 19A9, that specifically recognizes monomeric SOD1 while retaining the overall subunit scaffold. While monomerization has been proposed to facilitate SOD1 misfolding and aggregation in previous studies (Khare et al., 2004, 2006; Lindberg et al., 2005), our data do not allow us to determine whether the 19A9‐recognized species represents such an on‐pathway intermediate or directly contributes to neurotoxicity. Nonetheless, immunohistochemical analysis using 19A9 detected the folded SOD1 monomers in a subset of spinal motoneurons from DM‐affected dogs but not in healthy controls, suggesting that such monomeric species may be associated with protein aggregation and disease.

Although immunoreactivity with 19A9 was detected in spinal motoneurons of DM‐affected dogs expressing SOD1^E40K^, the E40K substitution had little effect on the crosslinking of SOD1 with 19A9^Fv‐c^ (Figure 1c), indicating that this substitution does not markedly enhance SOD1 monomerization. Our previous study demonstrated that the E40K substitution in canine SOD1 increases the solvent exposure of the Cys7 side chain located near the dimer interface, suggesting local perturbation of the interface (Shino et al., 2024). In the crystal structure of canine SOD1, Cys7 is normally buried within a hydrophobic core of the β‐barrel (Hashimoto et al., 2023). Notably, this core is imperfectly packed and contains a small cavity, rendering it particularly susceptible to perturbation. The E40K substitution is expected to compromise the conformational integrity of the β‐plug, a structural element that buttresses the β‐barrel and helps maintain this core. Destabilization of the β‐plug would be transmitted to the hydrophobic core, facilitating solvent exposure of Cys7 and thereby promoting local perturbation of the dimer interface and increasing the propensity of SOD1 to undergo monomerization.

The monomer‐dimer equilibrium of SOD1 is strongly influenced by its metal‐binding and thiol‐disulfide states (Arnesano et al., 2004), raising the possibility that the E40K substitution promotes monomerization by impairing metal‐binding and/or disulfide formation. Even in the disulfide‐bonded state, an increased propensity for monomerization has been proposed for a subset of ALS‐associated human SOD1 variants (Khare et al., 2004, 2006; Lindberg et al., 2005). In particular, folding and unfolding kinetics of disulfide‐intact human SOD1 have been analyzed in vitro using a three‐state model, in which the native homodimer first dissociates into monomers that subsequently unfold. Detailed analysis of those kinetics indicated that ALS‐associated mutations selectively destabilize the monomeric state, enhance dimer dissociation, or both (Lindberg et al., 2005). We also reported that monomerization of human SOD1 increases the susceptibility of Cys6 (corresponding to Cys7 in canine SOD1)—located near the dimer interface and normally buried within the protein—to oxidation (Yoshida et al., 2025). Oxidation of this buried residue enhances its hydrophilicity, thereby disrupting the hydrophobic core of SOD1 and ultimately leading to protein unfolding. Taken together, these observations support a model in which monomeric SOD1 serves as an on‐pathway intermediate linking the native dimer to misfolded and aggregated states.

Consistent with the notion that monomeric SOD1 represents a key intermediate, its presence has also been demonstrated in cellular and in vivo contexts. We previously developed a monobody that selectively recognizes monomeric SOD1 but not the native homodimer (Amesaka et al., 2024). Using this monobody, chemical crosslinking experiments detected a disulfide‐intact monomeric fraction for a subset of ALS‐causing human SOD1 mutants, but not for wild‐type SOD1, when these proteins were overexpressed in *Saccharomyces cerevisiae* and *Escherichia coli*. Monomeric SOD1 has also been detected in transgenic mouse models overexpressing human SOD1 with ALS‐associated mutations, including models expressing wild‐type SOD1 (Zetterstrom et al., 2007). In particular, in transgenic mice expressing SOD1 with the G85R substitution, the mutant protein was eluted predominantly as a monomer in size‐exclusion chromatography. These observations indicate that a subset of ALS‐associated SOD1 mutants exhibits an increased propensity for monomer formation, a species that may predispose the protein to misfolding and subsequent aggregation.

It should be noted that monomeric SOD1 has been examined in mouse models and human tissues using a polyclonal antibody known as the SEDI antibody (Rakhit et al., 2007). This antibody was generated by immunizing a rabbit with residues 143–151 of human SOD1, a segment that spans part of the dimer interface and is therefore normally buried and inaccessible as an epitope, but becomes exposed upon monomerization. While the SEDI antibody stained inclusion bodies in motoneurons of ALS patients carrying SOD1 mutations, it also recognized SOD1 denatured by urea or oxidation (Rakhit et al., 2007). It therefore remains obscure whether SEDI detects monomeric SOD1 that retains a subunit‐like three‐dimensional architecture or extensively unfolded forms.

In addition, several monoclonal antibodies recognizing non‐native conformations of SOD1 have been reported (Furukawa & Tokuda, 2020; Rotunno & Bosco, 2013). For those antibodies, however, not only are structures of their complexes with SOD1 unavailable, but even their sequence information has not been disclosed, which precludes detailed structural characterization of their epitopes. Indeed, structural information on SOD1‐antibody complexes remains extremely limited; to our knowledge, only four such structures have been reported to date: the crystal structure of canine SOD1^E40K^ in complex with the monoclonal antibody 22E1 from our previous study (Shino et al., 2024), and the crystal structures of native human SOD1 in complex with three distinct nanobodies (Cheng et al., 2025). To gain insight into SOD1 conformations in vivo, it will be important to determine the structures of SOD1‐antibody complexes and to accumulate structural information on their epitopes.

Taken together with our immunohistochemical data, the 19A9–SOD1 complex structure supports the presence of a monomeric SOD1 species in diseased motoneurons. More broadly, our work illustrates how structure‐guided epitope characterization can translate the functional specificity of conformation‐selective antibodies into concrete structural information. Together with the present structural characterization of the 19A9‐SOD1 monomer interaction, the continued development of a structurally annotated panel of conformation‐specific antibodies spanning native dimer, native‐like monomer, and misfolded or aggregated states will provide a rigorous framework for identifying abnormal SOD1 conformers present in ALS and DM tissues.

## 4 MATERIALS AND METHODS

### 4.1 Preparation of recombinant proteins

SOD1 proteins were prepared and purified as described previously (Shino et al., 2024). The N‐terminally acetylated SOD1 was prepared by co‐expression of the fission yeast NatB acetylation complex in *E. coli* (Johnson et al., 2010). The generation of monoclonal antibodies was also reported in our earlier work (Kobatake et al., 2017), and the 19A9 clone obtained in that study was selected for further analysis here. The amino acid sequences of the V_H_ and V_L_ regions of the clone 19A9 (mouse immunoglobulin G1 (IgG1), κ isotype) were determined (Syd Labs, USA) and analyzed using IMGT/V‐QUEST to identify the V‐J regions and CDRs of both chains. The identified sequences (Figure 1b) were then used for recombinant protein expression described below. Amino acid residues were numbered starting from the first residue of the V‐J region of each V_H_ and V_L_ chain, which was designated as position 1.

For the preparation of 19A9^Fv‐c^, pET15b plasmid vectors expressing the V_H_ and V_L_ domains of the clone 22E1 antibody, each C‐terminally fused to the SARAH domain (Shino et al., 2024), were modified by replacing the complementary DNAs (cDNAs) corresponding to the V_H_ and V_L_ domains of 22E1 with those of 19A9 using the In‐Fusion PCR method. To introduce a disulfide bond between V_H_‐SARAH and V_L_‐SARAH of 19A9, serine residues (Ser123 in V_H_‐SARAH and Ser146 in V_L_‐SARAH) were substituted with cysteine by the inverse PCR method. Expression of 19A9 V_H_‐SARAH and V_L_‐SARAH in *E. coli* BL21(DE3), reconstitution into 19A9^Fv‐c^, and purification were performed as described previously (Shino et al., 2024). The concentration of 19A9^Fv‐c^ was determined spectroscopically from the absorbance at 280 nm using an extinction coefficient of 60,195 M^−1^ cm^−1^.

For preparation of 19A9^Tri^, the cDNAs encoding the V_H_ and V_L_ regions of 19A9 were directly linked without a linker and cloned between the *Nco*I and *Sal*I sites of a modified pET‐15b plasmid (Furukawa et al., 2010). Amino acid residues were numbered sequentially from the V_H_ through to the V_L_ region. *E. coli* BL21(DE3) cells were transformed, and expression was induced with 0.5 mM isopropyl‐β‐D‐thiogalactopyranoside at 37°C for 5 h. The expressed protein accumulated as inclusion bodies, which were washed three times by suspension in phosphate‐buffered saline (PBS) containing 2% Triton X‐100, sonication, and centrifugation at 20,000 × *g*. Washed inclusion bodies were solubilized in 50 mM Tris, 150 mM NaCl, 3 M guanidine hydrochloride, and 10 mM β‐mercaptoethanol (β‐ME) (pH 8.0), diluted to ~100 μM with the same buffer, and incubated at 37°C for 30 min. The samples were dialyzed against 50 mM Tris, 500 mM NaCl (pH 8.0) at 4°C, and centrifuged to remove precipitates. Ammonium sulfate was added to the supernatant to 20% (w/v), followed by centrifugation at 20,000 × *g*, and the resulting supernatant was applied to a HiTrap Phenyl FF (high sub) column (Cytiva). 19A9^Tri^ was eluted with 10 mM Tris, 150 mM NaCl (pH 8.0). The eluate was buffer‐exchanged into 10 mM Tris (pH 8.0), loaded onto a HiTrap Q FF column (Cytiva), and eluted with 10 mM Tris, 30 mM NaCl (pH 8.0). The protein was further buffer‐exchanged into 50 mM 3‐(N‐morpholino)propanesulfonic acid (MOPS)roo, 100 mM NaCl (pH 7.0; MN buffer), and purified by gel‐filtration chromatography on a COSMOSIL 5Diol‐300‐II column (Nacalai Tesque). Fractions containing 19A9^Tri^ were pooled, concentrated, and stored at −80°C. The concentration of 19A9^Tri^ was determined spectroscopically from absorbance at 280 nm using a molar extinction coefficient of 43,110 M^−1^ cm^−1^.

The recombinant mouse 19A9 antibody was prepared following the procedure described in our previous study (Kosuge et al., 2023). Briefly, the cDNAs encoding the V_H_ and V_L_ domains of 19A9 were inserted into the corresponding regions of pcDNA3.4 expression vectors (Thermo Fisher Scientific) carrying the mouse IgG1 heavy chain and kappa light chain, respectively. Expi293F cells (Thermo Fisher Scientific) were transfected with those plasmids and cultured at 37°C under 8% CO_2_ for 5 days. The supernatant was collected through centrifugation of the cell culture for 10 min at 1000 × *g*. The recombinant 19A9 IgG was purified via affinity chromatography using rProtein A Sepharose Fast Flow (Cytiva) and subsequently via size‐exclusion chromatography using HiLoad 16/600 Superdex 200 pg column (Cytiva) equilibrated with PBS. The purified 19A9 antibody was quantified based upon the absorbance at 280 nm with the extinction coefficient of 108,750 M^−1^ cm^−1^.

### 4.2 Chemical crosslinking

Equimolar mixtures of SOD1 variants and 19A9^Fv‐c^ (20 μM each) in the MN buffer were incubated with the chemical crosslinker bis(sulfosuccinimidyl)suberate disodium (DOJINDO) at a final concentration of 2 mM. After an hour at room temperature, the reaction was quenched with 1 M Tris (pH 8.0) to a final concentration of 100 mM. Samples were then mixed with Laemmli sample buffer containing β‐ME, resolved by SDS‐PAGE on 15% polyacrylamide gels, and visualized by Coomassie Brilliant Blue R‐250 staining.

### 4.3 Size‐exclusion chromatography

Equimolar mixtures of SOD1 and 19A9 variants (10 μM each) were prepared in the MN buffer and analyzed by size‐exclusion chromatography on a COSMOSIL 5Diol‐300‐II column at a flow rate of 0.5 mL/min. Elution was monitored at 280 nm; however, because SOD1 variants have a very low molar extinction coefficient (1,615 M^−1^ cm^−1^), the profiles predominantly reflected the 19A9 variants. To visualize the elution behavior of both SOD1 and 19A9, eluates were therefore collected at 1‐min intervals and analyzed by SDS‐PAGE. Fractions were mixed with Laemmli sample buffer containing either 33 mM iodoacetamide and run under non‐reducing conditions (for 19A9^Fv‐c^, to preserve the disulfide‐linked V_H_‐SARAH and V_L_‐SARAH and thus avoid overlap with SOD1 of similar molecular weight) or 5.6% β‐ME (for 19A9^Tri^ and 19A9 antibody). Proteins were separated on 15% polyacrylamide gels and visualized with Coomassie Brilliant Blue R‐250.

### 4.4 Cryo‐electron microscopy data collection

19A9^Tri^ was mixed with SOD1^FG^ (supplemented with 1 equivalent each of CuSO_4_ and ZnSO_4_) at a 1:1.5 molar ratio and separated on a COSMOSIL 5Diol‐300‐II column. Fractions containing both proteins were pooled, buffer‐exchanged into 200 mM NaCl, 10 mM Tris–HCl (pH 8.0), and adjusted to 10 mg/mL. To improve particle distribution, 1.0 mM fluorinated Fos‐Choline‐8 was further added before freezing.

Quantifoil R1.2/1.3 Cu 300 mesh grids were glow‐discharged (10 s, 7 Pa, 10 mA; JEC‐3000FC, JEOL). Four microliters of sample were applied, blotted for 3.5 s (force 10) using a Vitrobot Mark IV (Thermo Fisher) at 8°C and 100% humidity, and plunge‐frozen in liquid ethane. Data were acquired on a CRYO ARM 300 microscope (JEOL, SPring‐8EM01CT) at 300 kV, equipped with an omega in‐column energy filter and a Gatan K3 detector. Movies were recorded in correlated double sampling (CDS) counting mode with SerialEM at a nominal magnification of 60,000× (0.752 Å/pixel), 60 frames per 3.11 s exposure (50 e^−^/Å^2^), using a 5 × 5 image shift matrix and a defocus range of −0.1 to −3.5 μm. In total, 6200 movies were collected.

### 4.5 Image processing, model building, and refinement

Data were processed using cryoSPARC v4.4 (Punjani et al., 2017), as summarized in Figure S3. Motion correction (Patch Motion) and contrast transfer function (CTF) estimation (Patch CTF) were applied, and 6,132 micrographs passed curation. From 500 micrographs, ~36,000 particles were initially picked and classified, generating triangular 2D templates. Iterative template‐based picking revealed two conformations (type A and B). For type A, ~423,000 particles were extracted, refined through successive 2D/3D classifications, and reduced to ~143,000 high‐quality particles. Refinement under C3 symmetry yielded a 2.95 Å map (FSC = 0.143). For type B, ~316,000 particles were extracted and processed to ~61,000 high‐quality particles, yielding a 3.91 Å map (FSC = 0.143). Local resolution was estimated in cryoSPARC. Maps were deposited in Electron Microscopy Data Bank (EMDB) (accession codes: EMDB‐67612 and EMDB‐67613 for type A and B, respectively).

Initial models of the V_H_–V_L_ complex of 19A9 and SOD1^FG^ were generated separately with ColabFold and manually docked into the density map of the type A complex using the Fit in Map tool of ChimeraX. V_H_ and V_L_ chain assignments and residue numbering were modified to reflect the fused Triabody architecture, such that each V_L_ domain was renumbered to follow the C‐terminus of its corresponding V_H_ domain. Models were refined by real‐space refinement in PHENIX (Afonine et al., 2018; Liebschner et al., 2019) with manual adjustments in Coot (Emsley & Cowtan, 2004), iterated until convergence. Validation with MolProbity (Chen et al., 2010) indicated 94.66% residues in favored regions, 4.55% in allowed regions, and 0.79% outliers.

The refined model for type A was used as the basis for type B: a V_H_–V_L_/SOD1^FG^ unit was fitted into the map of type B, adjusted, and refined as above. Data collection and refinement statistics are summarized in Table S3. Coordinates have been deposited in the Protein Data Bank (PDB ID: 21EN and 21EO for type A and B, respectively).

### 4.6 Immunofluorescence staining

Spinal cord tissues from PWC with DM, homozygous for the E40K mutation in the SOD1 gene, were used. All DM‐affected dogs were owned by their respective owners and had been diagnosed with DM by post‐mortem examination and histopathological analysis with owner's permission at the Gifu University Veterinary Teaching Hospital. Spinal cord tissues from control Beagle dogs (all wild‐type homozygous), exhibiting no neurological signs, were also used. Further details regarding the dogs used in this study are summarized in Table S2.

Cervical, thoracic, and lumbar spinal cord tissues were embedded in OCT compound (Leica) and sliced into 10 μm thick frozen sections. These sections were fixed in 4% paraformaldehyde (in PBS) for 15 min. To eliminate lipofuscin autofluorescence, the sections were immersed in TrueBlack® Lipofuscin Autofluorescence Quencher (Biotium, USA) (1:20 diluted in ethanol) for 30 s. The sections were then blocked by incubating them in 1% bovine serum albumin in PBS for 1 h at room temperature and incubated overnight at 4°C with either recombinant 19A9 antibody (10 μg/mL) or rabbit polyclonal anti‐SOD1 antibody (SOD‐100, Enzo Life Sciences, 1:100 dilution). These sections were then incubated for 1 h at room temperature with the corresponding secondary antibodies: goat anti‐mouse IgG conjugated with Alexa Fluor 594 (1:500) for recombinant 19A9 and goat anti‐rabbit IgG conjugated with Alexa Fluor 488 (1:200) for SOD‐100. After nuclear staining with 6‐diamidino‐2‐phenylindole (DAPI) (DOJINDO, 1:1,000 dilution), coverslips were placed on slides using Fluoromount/Plus (Diagnostic BioSystems, USA). The sections were observed under a laser scanning confocal microscope (LSM 900, Carl Zeiss).

## AUTHOR CONTRIBUTIONS

**YS** performed the experiments and analyses, including cryo‐electron microscopy structure determination, and wrote the manuscript. **NM** contributed to cryo‐electron microscopy data analysis and structural interpretation. **YK** performed immunohistochemical analyses using canine tissues and generated monoclonal antibodies. **HKa** generated monoclonal antibodies. **HKo**, **MN**, and **KT** produced and purified recombinant antibodies. **YF** conceived and supervised the study, acquired funding, and wrote the manuscript. All authors reviewed and approved the final manuscript.

## CONFLICT OF INTEREST STATEMENT

The authors declare no conflicts of interest.

## Supporting information


**Data S1.** Supporting Information.

## Data Availability

The data that support the findings of this study are available from the corresponding author upon reasonable request.
