# Supplementary material for: Structural analysis of Cu/Zn‐superoxide dismutase linked to neurodegenerative disease by antibody‐guided cryo‐EM
Source: Protein Sci. 2026 May 5;35(6):e70615. doi: 10.1002/pro.70615 (PMC13142091; doi:10.1002/pro.70615)
Supplement: Supplementary file 1 — Data S1. Supporting Information. [file PRO-35-e70615-s001.pdf]

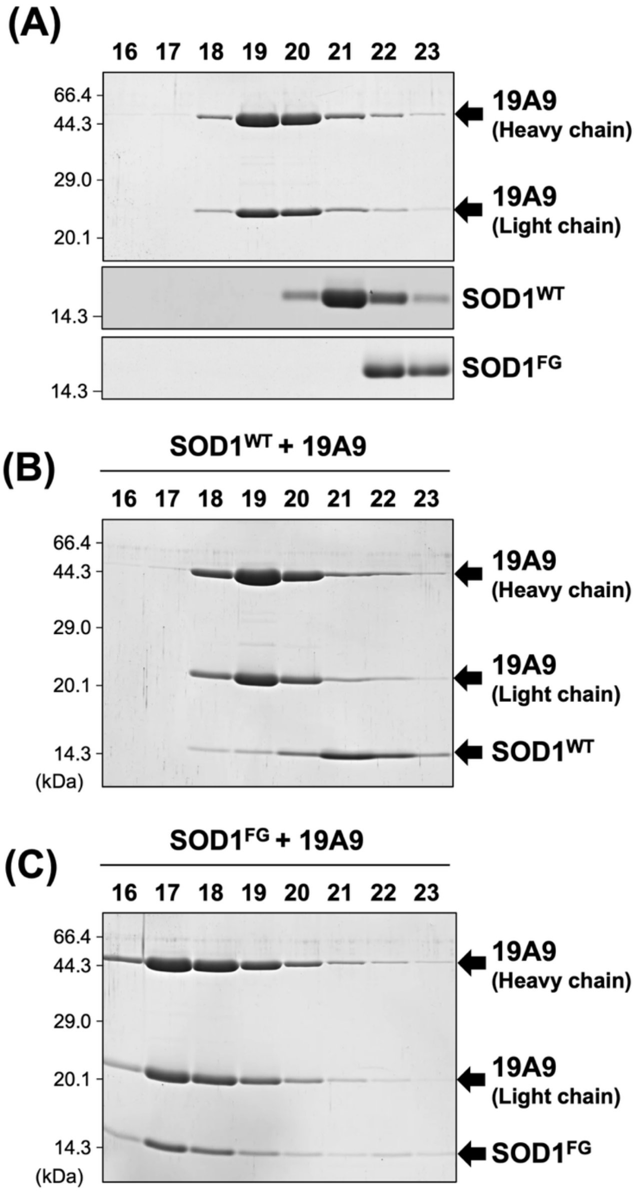

Figure S1 Examination of full-length 19A9 IgG binding to monomeric SOD1 by size-exclusion chromatography **(A)** Individual proteins (5 μM recombinant 19A9 IgG, 10 μM holo-SOD1^{WT}, and 10 μM holo-SOD1^{FG}) were analyzed by gel-filtration. The numbers indicated above each lane represent the fraction numbers, which correspond directly to the elution time (in minutes), as fractions were collected at 1-min intervals. Fractions 16-23 were examined by non-reducing SDS-PAGE followed by Coomassie Brilliant Blue staining. 19A9 (heavy and light chains) and holo-SOD1^{FG} were individually eluted at fraction numbers 19/20 and 22/23, respectively. **(B, C)** Recombinant 19A9 IgG (5 μM) was mixed with 10 μM holo-SOD1^{WT} (B) or 10 μM holo-SOD1^{FG} (C) and analyzed as described in (A). The results for holo-SOD1^{WT} and holo-SOD1^{FG} in Figure 2A are shown again in (A) as controls for (B) and (C). When a mixture of 19A9 IgG and holo-SOD1^{FG} was analyzed, both proteins were co-eluted earlier than when run separately, appearing together at fraction numbers 17/18 (C). This shift indicates complex formation between SOD1^{FG} and 19A9 IgG. In contrast, the elution profiles of holo-SOD1^{WT} and 19A9 IgG remained unchanged when mixed, suggesting that 19A9 IgG does not bind to dimeric SOD1 (B).

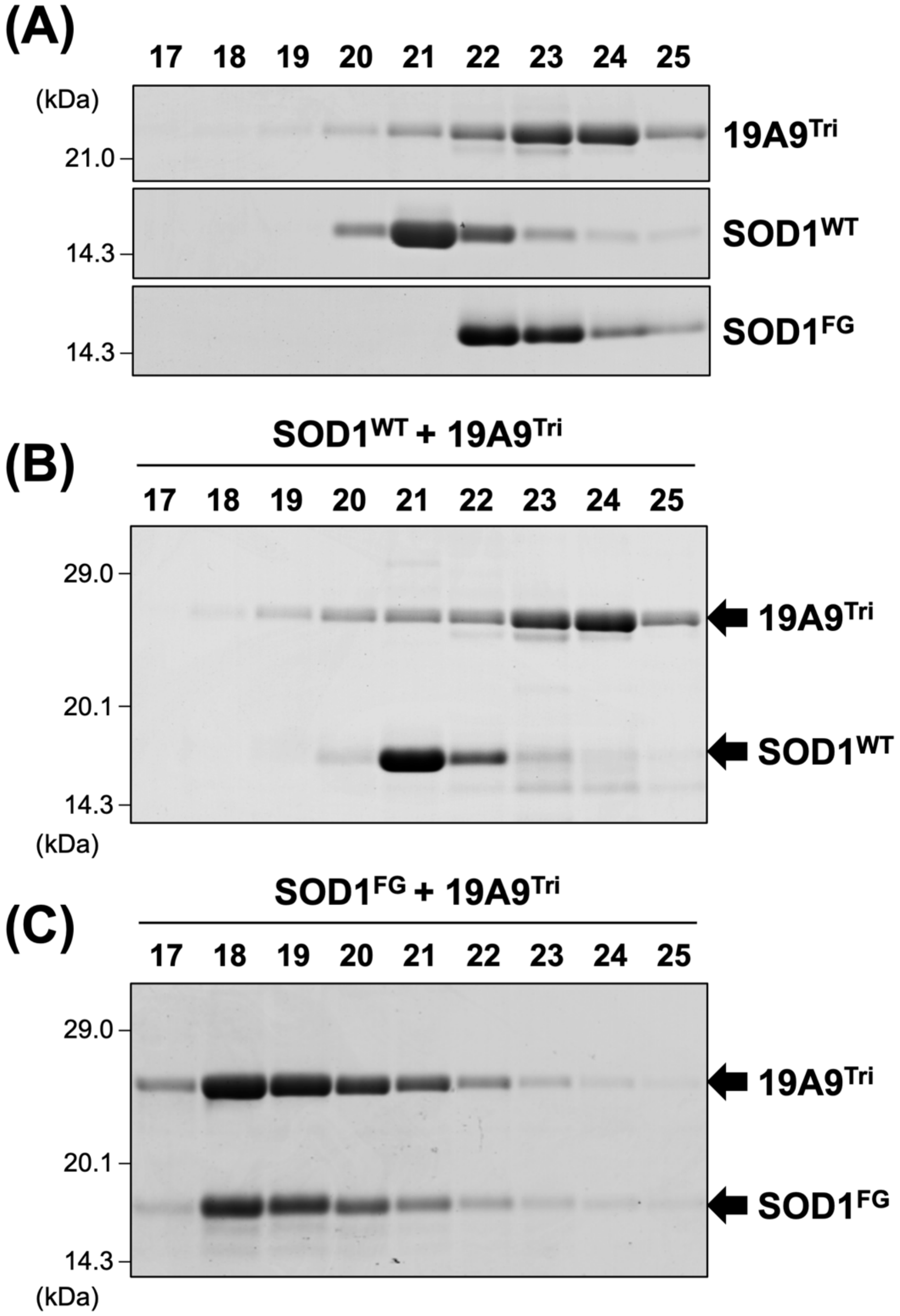

Figure S2 Examination of 19A9^{Tri} binding to monomeric SOD1 by size-exclusion chromatography **(A)** Individual proteins (19A9^{Tri}, holo-SOD1^{WT}, and holo-SOD1^{FG}; 10 μM) were analyzed by gel-filtration. The numbers indicated above each lane represent the fraction numbers, which correspond directly to the elution time (in minutes), as fractions were collected at 1-min intervals. Fractions 17-25 were examined by non-reducing SDS-PAGE followed by Coomassie Brilliant Blue staining. The results for holo-SOD1^{WT} and holo-SOD1^{FG} in Figure 2A are shown again in (A) as controls for (B) and (C). 19A9^{Tri} (10 μM) was mixed with equimolar **(B)** holo-SOD1^{WT} or **(C)** holo-SOD1^{FG} and analyzed as described in (A).

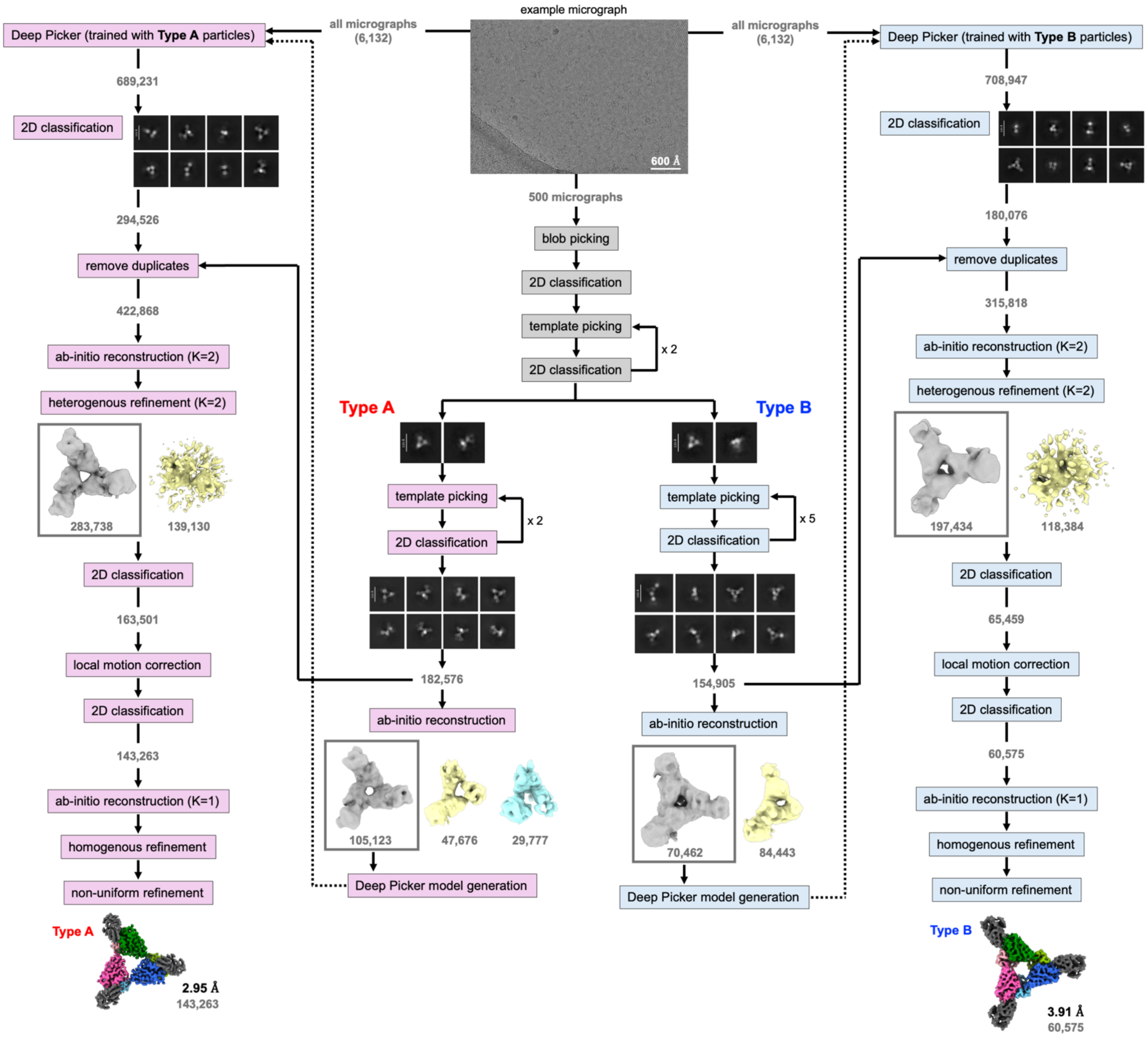

Figure S3 Workflow for cryo-EM data processing of the 19A9^{Tri} – SOD1^{FG} complex.

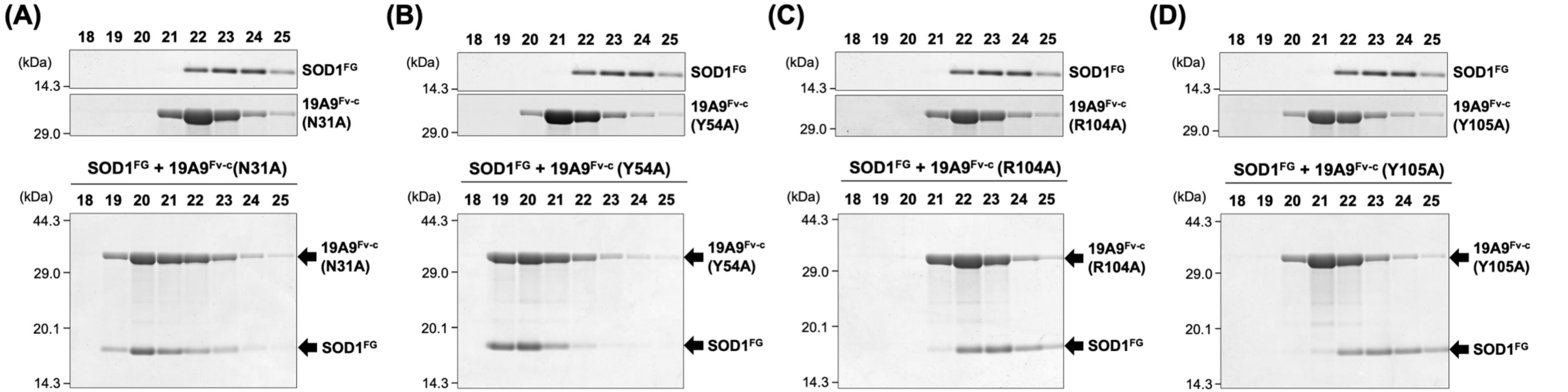

Figure S4 Examination of mutant 19A9$^{Fv\text{-}c}$ binding to monomeric SOD1 by size-exclusion chromatography **(A)** N31A, **(B)** Y54A, **(C)** R104A, and **(D)** Y105A variants of 19A9$^{Fv\text{-}c}$ were examined. (Upper panels) Individual proteins (mutant 19A9$^{Fv\text{-}c}$ and holo-SOD1^{FG}; 10 μM) were analyzed by gel-filtration. The numbers indicated above each lane represent the fraction numbers, which correspond directly to the elution time (in minutes), as fractions were collected at 1-min intervals. Fractions 18-25 were examined by non-reducing SDS-PAGE followed by Coomassie Brilliant Blue staining. The holo-SOD1^{FG} profiles in the upper panels are repeatedly shown as controls in (A-D). (Lower panels) Mutant 19A9$^{Fv\text{-}c}$ (10 μM) was mixed with equimolar holo-SOD1^{FG} and analyzed as described above.

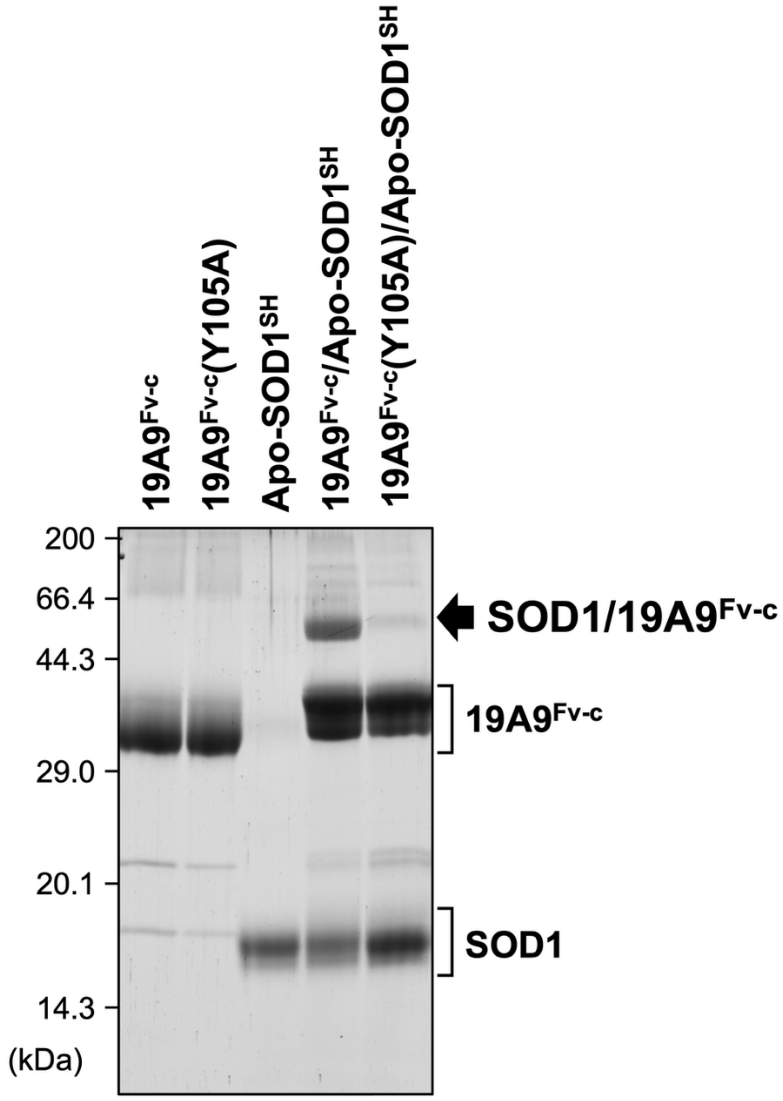

Figure S5 N-terminal acetylation of SOD1 does not affect recognition by 19A9 A cross-linking assay using the amine-reactive crosslinker bis(sulfosuccinimidyl)suberate was performed to examine the interaction of acetylated, disulfide-reduced apo-SOD1 in its monomeric state with $19A9^{Fv\text{-}c}$ and the Y105A variant of $19A9^{Fv\text{-}c}$. Non-reducing SDS-PAGE analysis revealed a band corresponding to acetylated SOD1 cross-linked with $19A9^{Fv\text{-}c}$ but not with the Y105A variant, indicating that N-terminal acetylation does not impair recognition of SOD1 monomer by 19A9.

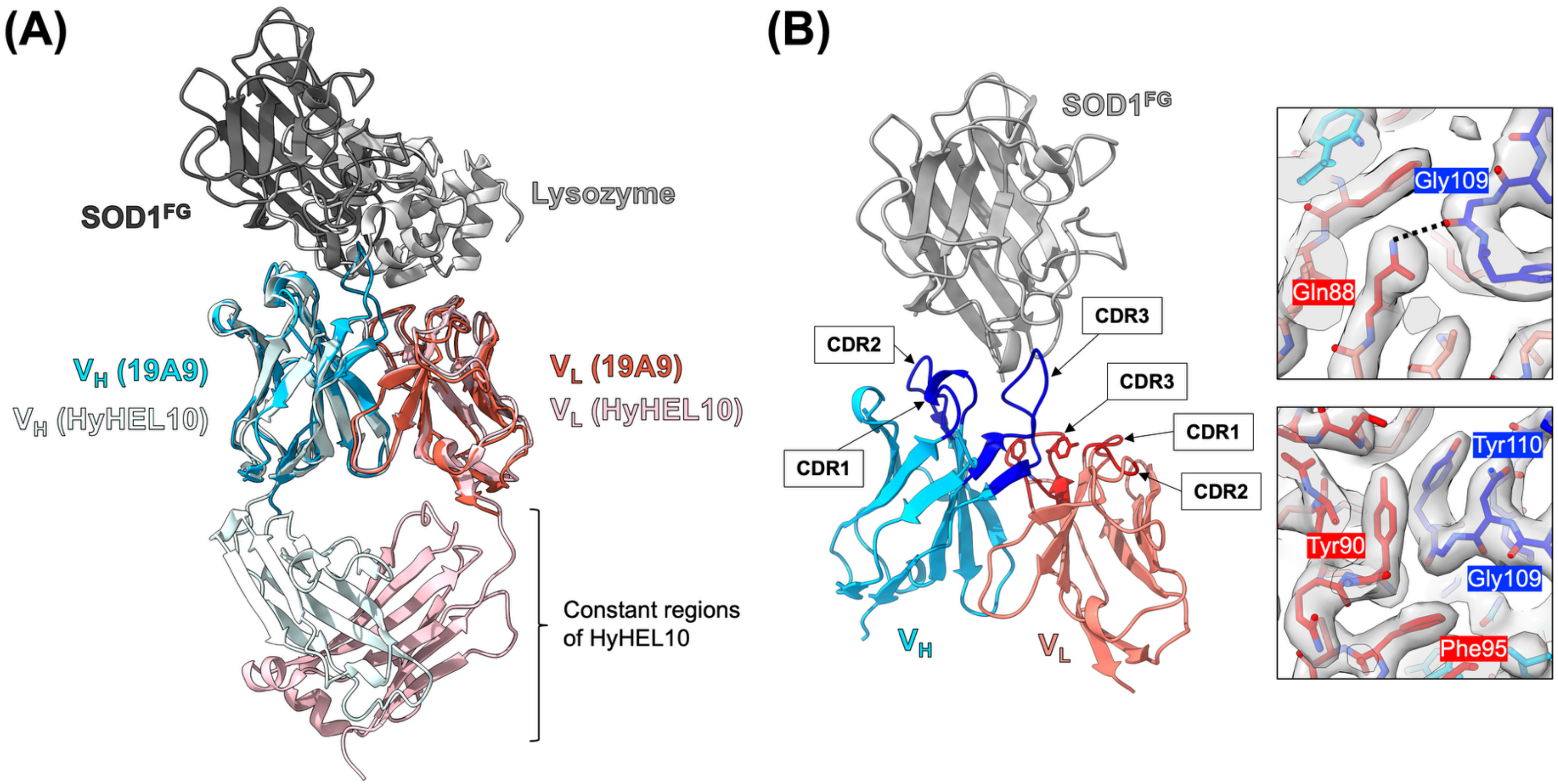

Figure S6 **Canonical configuration of V_H and V_L in 19A9** **(A)** The 19A9 V_H-V_L-SOD1^{FG} assembly is superimposed on the lysozyme-HyHEL10 complex by aligning the V_H-V_L regions. Coloring is as indicated in the figure. **(B)** (Left) Structural model of the 19A9 V_H-V_L-SOD1^{FG} complex, in which V_H (dark sky blue) and V_L (tomato) are shown with their three CDRs (CDR1-3) highlighted in blue and red, respectively. Residues within the CDRs of V_L that contact V_H are depicted as sticks. (Right) Close-up views of the interaction interface, highlighting the CDR3 of V_H (blue) and V_L (red). Key V_L residues (numbered in V_L-only numbering; Gln88, Tyr90, Phe95) and V_H residues (Gly109, Tyr110) are shown in stick representation, and the cryo-EM density map is displayed as a surface. Hydrogen bonds are indicated by dotted lines.

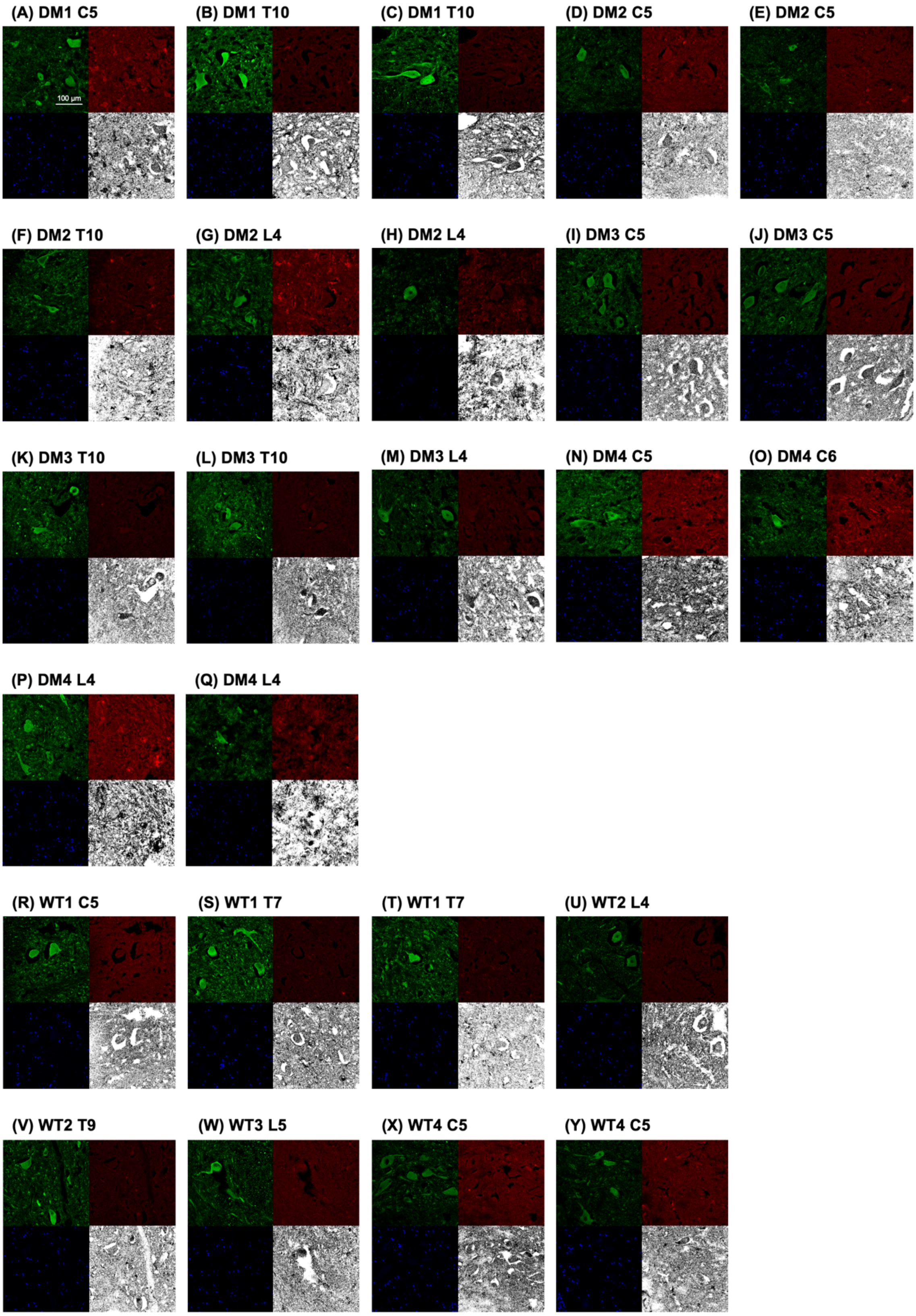
(A) DM1 C5
100 µm
(B) DM1 T10
(C) DM1 T10
(D) DM2 C5
(E) DM2 C5
(F) DM2 T10
(G) DM2 L4
(H) DM2 L4
(I) DM3 C5
(J) DM3 C5
(K) DM3 T10
(L) DM3 T10
(M) DM3 L4
(N) DM4 C5
(O) DM4 C6
(P) DM4 L4
(Q) DM4 L4
(R) WT1 C5
(S) WT1 T7
(T) WT1 T7
(U) WT2 L4
(V) WT2 T9
(W) WT3 L5
(X) WT4 C5
(Y) WT4 C5

Figure S7 Detection of monomeric SOD1 in motoneurons of DM-affected dogs by 19A9 Double fluorescence immunohistochemistry was performed on cervical (C5, C6), thoracic (T7, T9, T10), and lumbar (L4, L5) spinal cord sections. Shown are additional staining results from **(A-Q)** DM-affected Pembroke Welsh Corgis (E40K homozygotes, DM1-4) and **(R-Y)** asymptomatic Beagles (WT homozygotes, WT1-4). Details of the animals are provided in Supplemental Table S2. Representative images from DM1-3 and WT1-3 are presented in Figure 7, whereas this figure includes staining from different tissue sections of those same animals as well as sections from DM4 and WT4. Sections were co-stained with a polyclonal anti-SOD1 antibody (green) and recombinant monoclonal antibody 19A9 (red), with nuclei counterstained by DAPI (blue). Grayscale images in the lower-right corner of each panel display the 19A9 signal in the red channel after adjustment of the lower intensity threshold in ImageJ, which facilitated discrimination between stained and unstained cells.

Table S1 Hydrogen bond interactions between V_H of 19A9^{Tri} and SOD1^{FG} identified by PISA[1)]

Residue/Atom in V_H	Distance (Å)	Residue/Atom in SOD1^{FG}
**Side-chain (V_H) – Side chain (SOD1^{FG}) interactions**		
Tyr 32 (OH)	2.41	Asp 109 (OD1)
**Side-chain (V_H) – Main chain (SOD1^{FG}) interactions**		
Asn 31 (ND2)	2.97	Gly 108 (O)
Tyr 54 (OH)	3.43	Lys 4 (O)
Arg 104 (NH2)	2.79	Ser 26 (O)
Arg 104 (NH2)	2.74	Gly 27 (O)
Arg 104 (NH1)	2.42	Ile 104 (O)
Tyr 105 (OH)	2.40	Met 1 (N)
**Main-chain (V_H) – Side-chain (SOD1^{FG}) interactions**		
Asn 31 (O)	3.11	Ser 107 (OG)
Arg 104 (O)	3.34	Gln 23 (NE2)
Tyr 105 (O)	2.42	Ser 107 (OG)
**Main-chain (V_H) – Main-chain (SOD1^{FG}) interactions**		
Asn 31 (O)	2.90	Gly 108 (N)
Ser 102 (O)	3.24	Asp 109 (N)
Pro 103 (O)	3.46	Asp 109 (N)
Pro 103 (O)	2.98	Tyr 110 (N)

1) Hydrogen-bonding pairs with distances less than 3.50 Å were selected.

Table S2 Information on the dogs used in this study

	Breed	Age	Duration	*SOD1* Genotype	Sex	Postmortem interval
DM1	PWC[1)]	14y10m	3y	E40K homo	Male (castrated)	23 h
DM2	PWC[1)]	16y5m	3y	E40K homo	Male (castrated)	19 h
DM3	PWC[1)]	12y2m	3y7m	E40K homo	Male (castrated)	12 h
DM4	PWC[1)]	13y8m	2y10m	E40K homo	Male	23 h
WT1	Beagle	8y	---	WT	Female (sprayed)	0 h
WT2	Beagle	N.A.[2)]	---	WT	Female	0 h
WT3	Beagle	10y1m	---	WT	Female (sprayed)	0 h
WT4	Beagle	13y8m	---	WT	Female (sprayed)	0 h

1) Pembroke Welsh Corgi, 2) Not available

Table S3 Cryo-EM data collection, processing, refinement and validation statistics

	Type A (EMD-67612) (PDB ID: 21EN)	Type B (EMD-67613) (PDB ID: 21EO)
**Data collection**		
Microscope	CRYO ARM 300 (EM01CT at SPring-8)	
Accelerating voltage (kV)	300	
Electron source	CFEG	
Camera	K3	
Energy filter	In-column Ω filter	
Nominal magnification	60,000×	
Calibrated pixel size (Å)	0.752	
Defocus range (μm)	–0.10 to –3.5	
Total electron dose (e^{-}/Å^2)	59.66	
Number of frames	50	
Micrographs collected	6,200	
Micrographs used	6,132	
**Image processing**		
Initial particle images	422,868	315,818
Final particle images	143,263	60,575
Software used	cryoSPARC	cryoSPARC
Symmetry imposed	C3	C3
Map resolution (Å, FSC=0.143)	2.95	3.91
Map resolution range (Å)	1.6–46.7	3.3–58.6
Map sharpening B-factor (Å^2)	118.7	148.1
**Model refinement**		
Initial model used	ColabFold v1.5.5	refined type A model
Refinement software	PHENIX, Coot	PHENIX, Coot
Model resolution (Å, FSC=0.5)	3.37	4.38
Model-map CC (masked)	0.67	0.71
Model-map CC (box)	0.5	0.74
Model-map CC (peaks)	0.45	0.6
Model-map CC (volume)	0.67	0.7
**Model statistics**		
Chains	6	6
Atoms (hydrogens)	8691 (0)	8691 (0)
Residues	1155	1155
Water / Ligands	0 / 0	0 / 0
Bond length RMSD (Å)	0.020 (2 >4σ)	0.020 (0 >4σ)
Bond angle RMSD (°)	1.224 (11 >4σ)	1.535 (17 >4σ)
B-factors (min/max/mean, Å^2)	53.47 / 98.94 / 96.33	53.47 / 98.94 / 96.33
**Model validation**		
MolProbity score	1.78	2.21
Clashscore	7.71	19.1
Rotamer outliers (%)	0.85	0.11
Ramachandran plot (fav/allow/outl %, %)	94.75 / 4.46 / 0.79	93.35 / 5.86 / 0.79
Rama-Z (whole)	–1.54 (0.23)	–2.60 (0.22)
Rama-Z (helix / sheet / loop)	–5.36 / –0.82 / –0.98	–5.33 / –1.31 / –1.96
Cβ outliers (%)	0	0
CaBLAM outliers (%)	1.68	3.01
Peptide plane (cis / twisted)	6.2 / 0.0	6.2 / 0.0
Occupancy mean (%)	100	100
**Map statistics**		
Map min/max/mean	–2.98 / 4.76 / 0.02	–1.32 / 2.07 / 0.02
Box size (Å)	139.87 × 136.11 × 87.98	150.40 × 148.90 × 67.68
Supplied resolution (Å)	3	3.9
d99 (Å)	3.2	4.1
d(model) (Å)	3.2	4.1
d(FSC model 0/0.143/0.5)	2.8 / 2.9 / 3.3	3.7 / 3.8 / 4.2
